# Morphology, Connectivity, and Encoding Features of Tactile and Motor Representations of the Fingers in the Human Precentral and Postcentral Gyrus

**DOI:** 10.1523/JNEUROSCI.1976-21.2022

**Published:** 2023-03-01

**Authors:** Giulio Mastria, Eugenio Scaliti, Carsten Mehring, Etienne Burdet, Cristina Becchio, Andrea Serino, Michel Akselrod

**Affiliations:** ^1^MySpace Lab, Department of Clinical Neurosciences, University Hospital of Lausanne, University of Lausanne, Lausanne, CH-1011, Switzerland; ^2^C’MoN, Cognition, Motion and Neuroscience Unit, Fondazione Istituto Italiano di Tecnologia, Genova, 16163, Italy; ^3^Bernstein Center and Faculty of Biology, University of Freiburg, Freiburg, 79104, Germany; ^4^Department of Bioengineering, Imperial College of Science, Technology and Medicine, London, SW7 2AZ, United Kingdom

**Keywords:** body representation, fMRI, perception, polidactility, representational similarity analysis, sensorimotor

## Abstract

Despite the tight coupling between sensory and motor processing for fine manipulation in humans, it is not yet totally clear which specific properties of the fingers are mapped in the precentral and postcentral gyrus. We used fMRI to compare the morphology, connectivity, and encoding of the motor and tactile finger representations (FRs) in the precentral and postcentral gyrus of 25 5-fingered participants (8 females). Multivoxel pattern and structural and functional connectivity analyses demonstrated the existence of distinct motor and tactile FRs within both the precentral and postcentral gyrus, integrating finger-specific motor and tactile information. Using representational similarity analysis, we found that the motor and tactile FRs in the sensorimotor cortex were described by the perceived structure of the hand better than by the actual hand anatomy or other functional models (finger kinematics, muscles synergies). We then studied a polydactyly individual (i.e., with a congenital 6-fingered hand) showing superior manipulation abilities and divergent anatomic-functional hand properties. The perceived hand model was still the best model for tactile representations in the precentral and postcentral gyrus, while finger kinematics better described motor representations in the precentral gyrus. We suggest that, under normal conditions (i.e., in subjects with a standard hand anatomy), the sensorimotor representations of the 5 fingers in humans converge toward a model of perceived hand anatomy, deviating from the real hand structure, as the best synthesis between functional and structural features of the hand.

**SIGNIFICANCE STATEMENT** Distinct motor and tactile finger representations exist in both the precentral and postcentral gyrus, supported by a finger-specific pattern of anatomic and functional connectivity across modalities. At the representational level, finger representations reflect the perceived structure of the hand, which might result from an adapting process harmonizing (i.e., uniformizing) the encoding of hand function and structure in the precentral and postcentral gyrus. The same analyses performed in an extremely rare polydactyly subject showed that the emergence of such representational geometry is also found in neuromechanical variants with different hand anatomy and function. However, the harmonization process across the precentral and postcentral gyrus might not be possible because of divergent functional-structural properties of the hand and associated superior manipulation abilities.

## Introduction

The exceptional functionality of human fingers is unique in the animal kingdom and is likely one of the key factors contributing to the evolutionary advantage of the human species. Since seminal work by Penfield (Penfield and Boldrey, 1937), neurophysiological studies described dedicated representations for finger’s movements and somatosensation in the precentral and postcentral gyrus. With the advent of fMRI, primary sensorimotor representations of the fingers in humans have been extensively studied as a major model to investigate brain organization and plasticity (Makin et al., 2013, 2015; Bruurmijn et al., 2017; Hahamy et al., 2017; Serino et al., 2018).

Object manipulation requires the constant integration of motor commands and somatosensory information (Flanagan and Johansson, 2002; Johansson and Flanagan, 2009; Hatsopoulos and Suminski, 2011; Tamè et al., 2015; Turco et al., 2018). To date, most fMRI studies focused on either fingers’ motor or somatosensory function, while fingers’ motor and somatosensory representations have been rarely studied together by directly comparing brain activation induced by dedicated tasks (Wiestler et al., 2011; Berlot et al., 2019). Thus, the tight coupling between motor and somatosensory processing, although clearly stated, is often neglected in neuroimaging studies; and our understanding of the relationship between motor and somatosensory representations is scarce.

Here we investigate the processing of movements and touch within the precentral and postcentral gyrus to characterize the properties and the function of finger representations (FRs). To this aim, we used fMRI to map and compare the FRs recruited during two independent tasks, that is, fingers movement (motor FRs) and isolated tactile stimulation (tactile FRs), in the precentral and postcentral gyrus of the same participants. Based on anatomic (i.e., cortical distance and structural connectivity) and functional (i.e., pattern similarity and functional connectivity) properties, we analyzed the morphology of FRs and their representational geometry. This unique within-subject design allowed us to search for commonalities or dissociations between the representation of motor and tactile information in the precentral and postcentral gyrus.

We then investigated the “content” of motor and tactile FRs in the precentral and postcentral gyrus. Neurophysiological studies across different primates species suggest that different functional and neuroanatomical properties of the hand contribute to the FRs’ organization in the precentral and postcentral gyrus, and depend on the recruited modality (Krubitzer et al., 2004). Neuroimaging studies in humans showed that the dissimilarity between the activity patterns associated to each finger (termed representational geometry) is a reliable measure to compare FRs across cortical regions (Ejaz et al., 2015) or across modalities (Arbuckle et al., 2019; Berlot et al., 2019; Sanders et al., 2019), and can inform about the most likely features encoded by FRs (Ejaz et al., 2015; Akselrod et al., 2021; Tamè et al., 2021). A seminal study by Ejaz et al. (2015) demonstrated that, in both the precentral and postcentral gyrus, the co-occurrence of finger movements can describe the representational geometry of the fingers better than muscle synergies. Similarly, the anatomic structure of the hand has been also used to model tactile FRs in the postcentral gyrus (Akselrod et al., 2021). Recently, a study by Tamè et al. (2021) found that the differences in the cortical patterns elicited in the precentral and postcentral gyrus by tactile stimuli over the hand can be better described by their perceived rather than their actual location on the skin. However, these alternative models of hand representation (i.e., functional models based on kinematics and muscle synergies and structural models based on real and perceived anatomic features) have never been directly compared; and it is not clear whether the precentral and postcentral gyrus encodes similar or distinct properties of the hand, nor whether these can vary according to the represented modality, motor or tactile.

Considering that the relationship between hand structure, hand function, and cortical organization in humans has evolved to support dexterous capacities of hands with 5 fingers, it is possible that the human sensorimotor system is optimally organized for this specific hand architecture. Therefore, more complex anatomic variants (Mehring et al., 2019) may result in different cortical organizations (for extensive usage of 6-fingered hand orthosis, also see Kieliba et al., 2021). To approach this question, we had the extraordinary opportunity to test a congenital polydactyly individual with 6 fully developed fingers. Our previous work showed that the supernumerary finger provides enhanced motor capabilities compared with 5-fingered controls and has dedicated neural representations in the sensorimotor cortex (Mehring et al., 2019). Here we compared the representational geometry of FRs in the precentral and postcentral gyrus across motor and tactile tasks with models of hand function and structure obtained from this particular individual, with the same approach used on 5-fingered individuals. The extraordinary relationship between the structural and functional hand properties in this polydactyly individual provides a unique model to study how the human sensorimotor cortex self-organizes to represent different effectors’ architectures.

## Materials and Methods

Twenty-five healthy subjects participated in the study (8 females, mean age = 23.2 years, SD = 2.1 years). Compared with previous work, the study by Ejaz et al. (2015) used a sample of 12 “hands” (6 subjects × 2 hemispheres), which was sufficient to observe differences between three models (model of finger kinematics, model of muscle synergies, and model of somatotopy). With a probability of Type I error < 0.5 and a power > 0.8, with *n* = 50 (25 participants × 2 hemispheres), we estimated to have the statistical power to perform at least the same comparisons. Moreover, our sample size is larger than typically used in studies with similar design (Ejaz et al., 2015, *n* = 12; Berlot et al., 2019, *n* = 7; Tamè et al., 2021, *n* = 12).

We also used previously acquired data from a 17-year-old subject with 6 anatomically fully developed fingers on the two hands (see Mehring et al., 2019). All participants were right-handed and gave written informed consent to all procedures and data usage before the experiment started. The experimental procedures were approved by the Ethical Committee of Human Research of the Vaud canton (CER-Vd, project identifier: 2017-01588), Switzerland, and were conducted in accordance with the ethical guidelines of the ethical committee and the Declaration of Helsinki.

### Experimental procedure

Four fMRI runs were acquired in pseudo-randomized order across participants: for each hand separately, one run for the motor mapping and one run for the tactile mapping. In each run, the fingers of the same hand were either voluntarily moved by the participant (motor mapping) or received tactile stimulation (tactile mapping) in a fixed order (D1 - D3 - D5 - D2 - D4). The stimulation was repeated 6 times per finger. During the motor mapping, with the palmar side resting on a soft pad, subjects performed individuated finger extension movements at a rate of 1 Hz. Movement periods of 12 s were interleaved with periods of 8 s of rest (rest periods with no finger movement). Instructions (finger to move and movement pace) were projected on a mirror mounted onto the MR coil. The tactile stimulation elicited by the motor task was mainly on the palmar surface of the fingers. During the tactile mapping, subjects received tactile stimulation on the dorsal part of the two distal phalanges. The tactile stimulation was delivered by the experimenter by using a brush at a rate of 1 Hz, during stimulation periods of 12 s interleaved with periods of 8 s of rest. During the tactile stimulation, subjects were instructed to attend the tactile stimulation and to fixate a white cross on black background projected on the mounted mirror.

During the motor task, subjects received proprioceptive as well as tactile feedback. These sensory components would also contribute to the activity observed in the precentral and postcentral gyri and inflate the correspondence between the activity patterns associated with the two tasks. To increase the independence of the two tasks, we intentionally delivered touch on the dorsum of the fingers. It should be noted that the two tasks also differ in the quality of the tactile stimulation: while the rapid tactile stimulation of the motor task reaches the somatosensory cortex through myelinated A fibers, slow touch delivered during the tactile mapping is processed by both the somatosensory cortex and the insula though the activation of unmyelinated C fibers. Previous studies comparing active and passive finger movements showed a high degree of similarity between the tactile and motor FRs, but it is not clear whether this result was because of practically identical somatosensory stimulation during the two conditions (Berlot et al., 2019), or whether the motor and somatosensory processing have shared and converging representations.

### Image acquisition

MR images were acquired using a Siemens Prisma fit 3T MR with a 64-channel receiving headcoil. fMRI data, including four task-based runs (300 volumes each) and one resting-state run (150 volumes), were acquired using a multislice sequence (multislice factor = 2, GRAPPA = 3, TR = 2 s, TE = 0.03 s, 2 mm^3^ isotropic). Diffusion-weighted images (HARDI, 147 volumes and 10 b0 volumes, maximum b value 3000 s/mm^2^, 1.6 mm isotropic), and T1-weighted anatomic scans (3D MPRAGE sequence, 1 mm isotropic) were also acquired.

### Image preprocessing

Spatial realignment and normalization of fMRI data were conducted using the SPM12 software (Wellcome Department of Cognitive Neurology, London). Normalized images were minimally smoothed with an FWHM of 3 mm. Processing of the T1-weighted anatomic scans images was performed using the Connectome Mapper version 3.0.0-β-RC1 (https://connectome-mapper-3.readthedocs.io/en/latest/) (Tourbier et al., 2020). Gray and white matter was segmented and individual cortical surface reconstruction was performed using freesurfer from the MPRAGE volume (Desikan et al., 2006). Parcellation into 512 cortical and subcortical areas was performed according to the Lausanne anatomic atlas using the Connectome Mapper version 3.0.0-β-RC1 (Hagmann et al., 2008, 2010; Honey et al., 2009; Cammoun et al., 2012) by means of cortical surface-based registration to the high resolution T1-weighted anatomic scans. Correction for motion and eddy current distortions was performed on the HARDI sequences using FSL’s MCFLIRT and Eddy correct tool. Symmetric diffeomorphic SyN registration was performed from T1 to b0. The diffusion data were reconstructed with DSI studio using generalized q-sampling imaging (Yeh et al., 2010). The MRIcroGL software was used for visualizing results in 3D space for all subjects (McCausland Center for Brain Imaging, University of South Carolina) (https://www.mccauslandcenter.sc.edu/mricrogl/).

### GLMs

As part of the univariate analysis of the bold signal, a first-level GLM analysis (GLM1) was conducted in SPM on the normalized images The model included 5 regressors (one for each finger) convolved with the hemodynamic response and with the corresponding first-order time derivatives, as well as the 6 rigid-body motion parameters as nuisance regressors. The results of the first-level GLM1 were used to compute a second-level group GLM to identify common Hand ROIs for all subjects (see below). A similar GLM (GLM2) was computed on the preprocessed images in their native space to estimate subject’s individual responses to finger motion and touch. GLM2 was used to select the individual Finger ROIs (see below). In order to perform the analysis of multivoxel activity patterns, we computed a GLM (GLM3) analysis with 30 regressors and 6 rigid body motion regressors per run to estimate the β parameters associated with each period of tactile stimulation and finger movement.

### ROI selection

ROIs were defined for the hand (Hand ROIs, i.e., an area comprising voxels activated in relation to either of the fingers), and for each finger (Finger ROIs). Hand ROIs were used to perform multivoxel pattern analysis. Additionally, they were treated as masks to find finger-specific peaks of activation to define the Finger ROIs and to calculate the cortical volumes of activation of the FRs (see below). Finger ROIs were used to measure the Euclidean distances, as well as the functional and structural connectivity between individual fingers.

**Figure 1.**
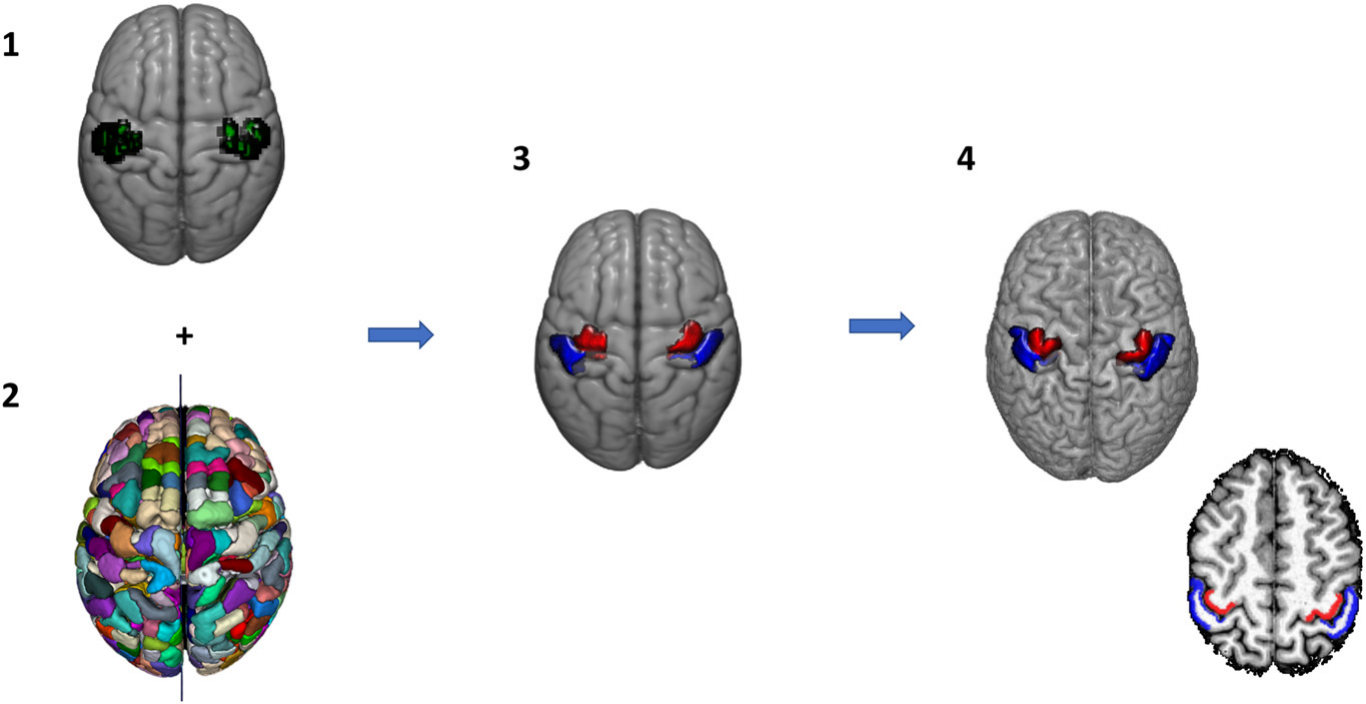
Flowchart of the Hand ROI selection. We first computed a group-level analysis in which we found clusters of voxels responding to motor and/or tactile stimulation of the fingers in MNI space (***1***). We then selected and fused, separately for the precentral and postcentral gyrus, the parcels of the Lausanne parcellation template in MNI space (***2***), which overlapped with active regions for >20% of their volume (***3***). We used the same parcels in the participants’ individual parcellation (in native space) as Hand ROIs (***4***).

**Figure 2.**
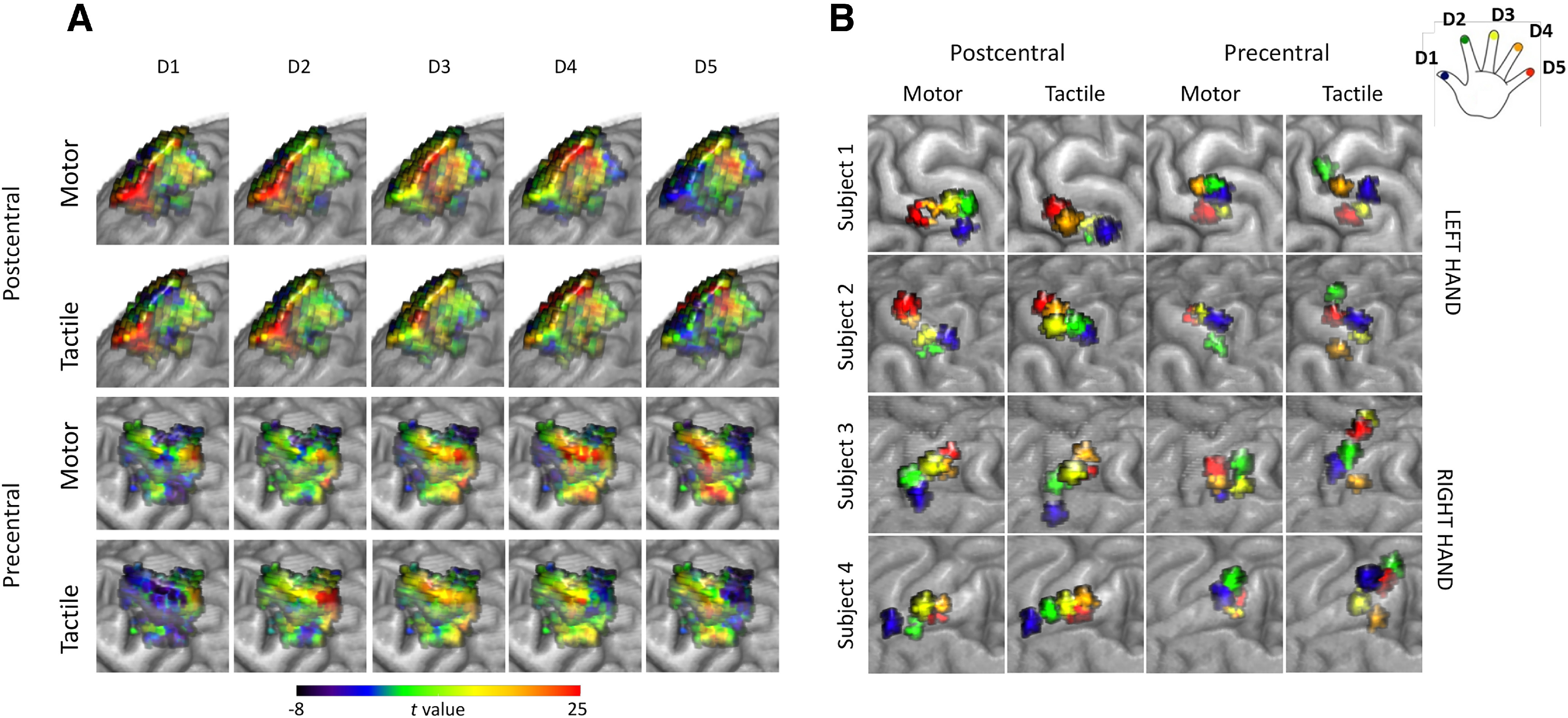
Hand ROIs for pattern extraction: t-maps of each finger versus rest in an example subject (***A***). Two top rows, Anteroposterior view of the Hand ROI in the postcentral gyrus. Two bottom rows, Posteroanterior view of the Hand ROI in the precentral gyrus. Finger ROIs: each map consists of a set of five ROIs defined around the finger-specific peaks of activity for 4 representative subjects (***B***).

For the Hand ROIs, we first defined a hand area in the precentral and postcentral gyrus of each hemisphere as follows. GLM1 was used to compute first-level t-contrasts comparing the activation of each finger versus rest, for the left and right hand of each subject, during the tactile and motor mapping. The first-level contrast images were entered in a second-level GLM to find voxels activated by the movement or the tactile stimulation of the fingers at the group level, in MNI space. At the group level, we first computed an F-contrast (*p* < 0.05 FWE cluster level corrected with *p* < 0.001 as cluster defining threshold) across all fingers, each finger versus rest (df = 5), for each hand and each task separately. The resulting clusters of activation were combined into a single ROI, which thus comprised voxel responding to either the movement and/or tactile stimulation of the fingers at the group level. Regions of the Lausanne parcellation, in the precentral and postcentral gyrus, overlapping for >20% with this ROI was selected in each hemisphere (Fig. 1). The corresponding regions of the Lausanne atlas in each subject’s individual parcellation (i.e., in native space) were selected and combined, separately for the precentral and postcentral gyrus, and used as ROIs defining the precentral and postcentral hand area in each hemisphere, with a total of 100 Hand ROIs (four Hand ROIs for each subject, Figs. 1 and 2).

The following analyses were al conducted in the native space of the subjects. For the Finger ROIs, separately for the motor and tactile tasks, a selective t-contrast (1 finger vs the remaining 4) was computed for each finger from GLM2 (individual GLM in native space). Using the Hand ROI as mask, finger specific ROIs were defined as spheres of 2 mm radius around the peak of activation (i.e., maximum positive *t* value) of each finger’s selective contrast (one finger vs all the other fingers), thus obtaining four finger maps in each hemisphere, that is, a total of 40 Finger ROIs for each subject (5 fingers × 2 tasks × 2 gyri × 2 hemispheres, Fig. 1*B*). The overlaps between the spheres were removed from the analysis. Of 1000 Finger ROIs being searched (40 ROIs × 25 subjects), only 35 were missing because of complete overlap or the absence of a positive *t* value. These Finger ROIs were used to measure the Euclidean distances and the functional connectivity between fingers.

A similar procedure was followed to define Finger ROIs in the white matter as seeding regions for the fiber tracking (i.e., structural connectivity). In this case, spherical ROIs of 2 mm radius were defined around the voxel in the white matter, which was closest to the peak of the finger selective t-contrast using GLM2. The overlaps between the spheres were removed from the analysis.

### Measures

#### Cortical volumes

Using GLM2, a t-contrast versus rest (*p* < 0.001) was used to compute the volume of activation of each finger during movement and tactile stimulation in each Hand ROI. The volume was calculated as the number of active voxels normalized for the total number of voxels of the individual Hand ROI. The cortical volume of activation can be interpreted as the size of the cortex dedicated to the representation of a finger, or in case of a movement, the spreading of the neural activity caused by the recruitment of that finger to perform a specific task.

#### Euclidean distances

Separately for the tactile and the motor representations in the precentral and postcentral gyrus, the Euclidean distances in 3D between pairs of Finger ROIs were calculated for each participant using the formula: $\sqrt[{2}]{{\left({\text{x}}_{2}-{\text{x}}_{1}\right)}^{2}+{\left({\text{y}}_{2}-{\text{y}}_{1}\right)}^{2}+{\left({\text{z}}_{2}-{\text{z}}_{1}\right)}^{2}}$ .

#### Multivoxel pattern dissimilarity

In order to study the representational geometry of FRs in the precentral and postcentral gyrus, we computed the cross-validated Mahalanobis distances between activity patterns obtained from GLM3 in the Hand ROIs (for a description of GLM3, see GLMs). For cross-validation, the 6 repetitions of the D1 to D5 stimulation sequence were split into even and odd number of repetitions, to form two partitions. In order to remove the effect induced by a different average activity in the tactile and motor conditions, which could bias the comparison between the pattern dissimilarity of the two conditions, we removed the average activity from each condition. We obtained 5 × 5 dissimilarity matrices for the motor FRs in the precentral and postcentral gyrus, and 5 × 5 dissimilarity matrices for the tactile FRs in the precentral and postcentral gyrus; 10 × 10 dissimilarity matrices in the precentral and postcentral gyrus were computed by analyzing the trials of both tasks together, which describe the similarity between FRs measured with different tasks in the same cortical areas (e.g., similarity between D1_tactile_ – D1_motor_ in the postcentral gyrus).

#### Structural connectivity

The structural connectivity between Finger ROIs in the white matter was estimated for individual participants using deterministic streamline tractography on reconstructed DSI data implemented in DSI studio (http://dsi-studio.labsolver.org). Within each voxel, the starting points were spatially random. The angular threshold was randomly selected from 15 to 90 degrees. A total of 200,000 tracts were calculated for each set of Finger ROIs. The structural connectivity between pairs of Finger ROIs was measured in terms of fiber density, defined as the number of streamlines between the two regions. In each matrix, the structural connectivity was normalized by the maximum fiber count, obtaining a measure of similarity between fingers.

#### Functional connectivity

The functional connectivity was computed for individual participants using the Conn toolbox (https://web.conn-toolbox.org/) on the resting state data. At each voxel, the BOLD signal was bandpass filtered (0.008-0.09 Hz). fMRI volumes were corrected for physiological variables, including regression of white matter, CSF, as well as motion (three translations and three rotations, estimated by rigid body coregistration in SPM12). The bivariate temporal correlations between pairs of Finger ROIs were calculated from the preprocessed BOLD time-courses of the resting state run. Before computing statistics, the coefficients were transformed into Gaussian values by applying the Fisher transform (Fisher, 1915).

### Statistical analysis

#### Analysis of the cortical volumes

Fifty hands (25 subjects × 2 hands) were included in the statistical analyses as independent observation. In order to test differences in the volume of activation, we computed a repeated-measures ANOVA including fingers (*finger*), areas (*gyrus*), and stimulation conditions (*task*) as factors (5 *finger* × 2 *gyrus* × 2 *task*), running *post hoc* comparison using paired *t* tests.

#### Analysis of somatotopy

Somatotopy is defined as a topographical arrangement on the cortical sheet of body part representations, which corresponds to their physical arrangement on the body. Therefore, to measure the degree of somatotopy of hand representation in each area and modality, we tested the hypothesis that the FRs were orderly organized in a serial arrangement from D1 to D5 (Penfield and Boldrey, 1937; Moore et al., 2000). To do so, data obtained from the analyses of cortical distance were statistically evaluated using Bayesian statistics (Akselrod et al., 2021). We used the R package *bain* (Hoijtink et al., 2019) to compute four Bayesian ANOVAs, one for each gyrus and modality. We compared three hypotheses: (1) H1, a hypothesis of equivalence between the tested variables with a difference between pairs of variables smaller than a Cohen’s *d* of 0.25 (D1-D2 ≈ D1-D3 ≈ D1-D4 ≈ D1-D5) (Sawilowsky, 2009); (2) H2, a hypothesis of ordering between the tested variables (D1-D2 < D1-D3 < D1-D4 < D1-D5); and (3) Hu (D1-D2, D1-D3, D1-D4, D1-D5), the alternative unrestricted hypothesis (corresponding to the null hypothesis). For all the ANOVAs we assumed equal prior probabilities and report the Bayes factors and posterior probabilities.

#### Pattern component modeling (PCM)

In order to investigate differences in the encoding structure of the tactile and motor FRs in the precentral and postcentral gyrus, we performed an encoding analysis using PCM (Diedrichsen and Kriegeskorte, 2017). The simple correlation between activity patterns usually underestimates their true correlation because of measurement noise. Further, differences in raw correlations can be biased by different levels of signal strength across area and experimental conditions. PCM can be used to address these issues by testing different models on the strength of the correlation between the finger-specific patterns. A full example of a PCM correlation model can be found online (https://pcm-toolbox-python.readthedocs.io/en/latest/demos/demo_correlation.html). We created 20 correlation models with correlations in the range [0.05, 1] in equal step sizes, namely, from a low correlation model where movement and tactile activity patterns are almost unrelated (*r* = 0.05) to a perfect correlation model in which the finger-specific patterns related to the tactile stimulation and the movement of the fingers are identical or a scaled version of each other (*r* = 1). We then calculated for each subject the log-likelihoods factor of the 20 correlation models and compared the log-likelihoods of the best fitting model to the log-likelihoods of all the other models using parametric statistics (paired *t* tests).

#### Multivoxel pattern analysis

In order to test differences in the level of discrimination between the FRs in the precentral and postcentral gyrus and in the motor and tactile modality, we compared the average pattern distance using a 2 × 2 repeated-measures ANOVA, including *task* and *gyrus* as factors.

We performed PCM to assess whether the tactile and motor FRs are likely to encode the same information. We then asked whether the activity patterns related to the two modalities are distinguishable, namely, whether there is a dissociation between motor and tactile FRs in the precentral and postcentral gyrus. We compared by comparing the dissimilarity between the activity patterns associated with adjacent fingers within each modality (e.g., D1_tactile_ – D2_tactile_) versus the dissimilarity between the activity patterns associated with the same finger across modalities (e.g., D1_tactile_ – D1_motor_). To this end, we calculated the mean of the elements adjacent to the diagonal in the bottom left and top right quadrants of the 10 × 10 multivoxel pattern dissimilarity matrices, thus obtaining the average dissimilarity between FRs within the motor and tactile modality, respectively. We then calculated the mean of the diagonal elements in the bottom right quadrant, obtaining the average dissimilarity between FRs across modalities. We entered the mean dissimilarity values in a repeated-measures ANOVA including modalities (*modality*: intratactile, intramotor, and intermodality) and areas *(gyrus*) as factors (three *modality* × 2 *gyrus*). *Post hoc* comparisons were conducted using paired *t* test.

We also compared the representational similarity across modality of the same finger (e.g., D1_tactile_ – D1_motor_) versus the adjacent fingers (e.g., D1_tactile_ – D2_motor_). A higher similarity across modality of the same finger compared with adjacent fingers could be expected in the postcentral gyrus, while the existence of such a finger-specific cross-modal organization could not be given for granted in the precentral gyrus, given the limited knowledge about the properties of the tactile FRs in this area. To conduct this analysis, we calculated the mean of the diagonal elements, obtaining the average dissimilarity between the representation of the same finger across modalities, and the mean of the elements adjacent to the diagonal in the bottom right quadrant, as the average dissimilarity between adjacent fingers across modality, of the 10 × 10 multivoxel pattern dissimilarity matrices. We then compared them using a repeated-measures ANOVA including *finger pairs* (same-finger and adjacent fingers) and areas (*gyrus*) as factors (2 *finger pairs* × 2 *gyrus*). *Post hoc* comparisons were conducted using paired *t* test on significant effects.

For display purposes, we used classical multidimensional scaling to represent dissimilarity matrices on 2D plots.

#### Analysis of the connectivity

We investigated the structural and functional connectivity between the motor and the tactile Finger ROIs in the precentral and postcentral gyrus. We conducted the same analysis described in Multivoxel pattern analysis using the 10 × 10 of functional and structural connectivity matrices. Since both the functional and structural connectivity are influenced by the cortical distance, we divided the connectivity matrices by the Euclidean distance matrices between ROIs of each subject to remove this bias.

#### Models of FRs

We compared different models of hand function and structure to determine which features are most likely encoded by FRs in the precentral and postcentral gyrus as a function of the motor or tactile condition.

Previous studies identified promising models explaining the features encoded by FRs. A study by Ejaz et al. (2015) demonstrated that the representational geometry of the fingers can be well modeled by the co-occurrence of finger movements in both the precentral and postcentral gyrus of the fingers better than muscle synergies. Similarly, the anatomic structure of the hand has been also used to model tactile FRs in the postcentral gyrus (Akselrod et al., 2021). Recently, a study by Tamè et al. (2021) found that the differences in the cortical patterns elicited in the precentral and postcentral gyrus by tactile stimuli over the hand can be better described by their perceived rather than their actual location on the skin. This finding is in a line with several studies demonstrating important and systematic distortions in hand perception, particularly in the perceived ratio between hand width and length (Longo and Haggard, 2010; Sadibolova et al., 2018).

**Figure 3.**
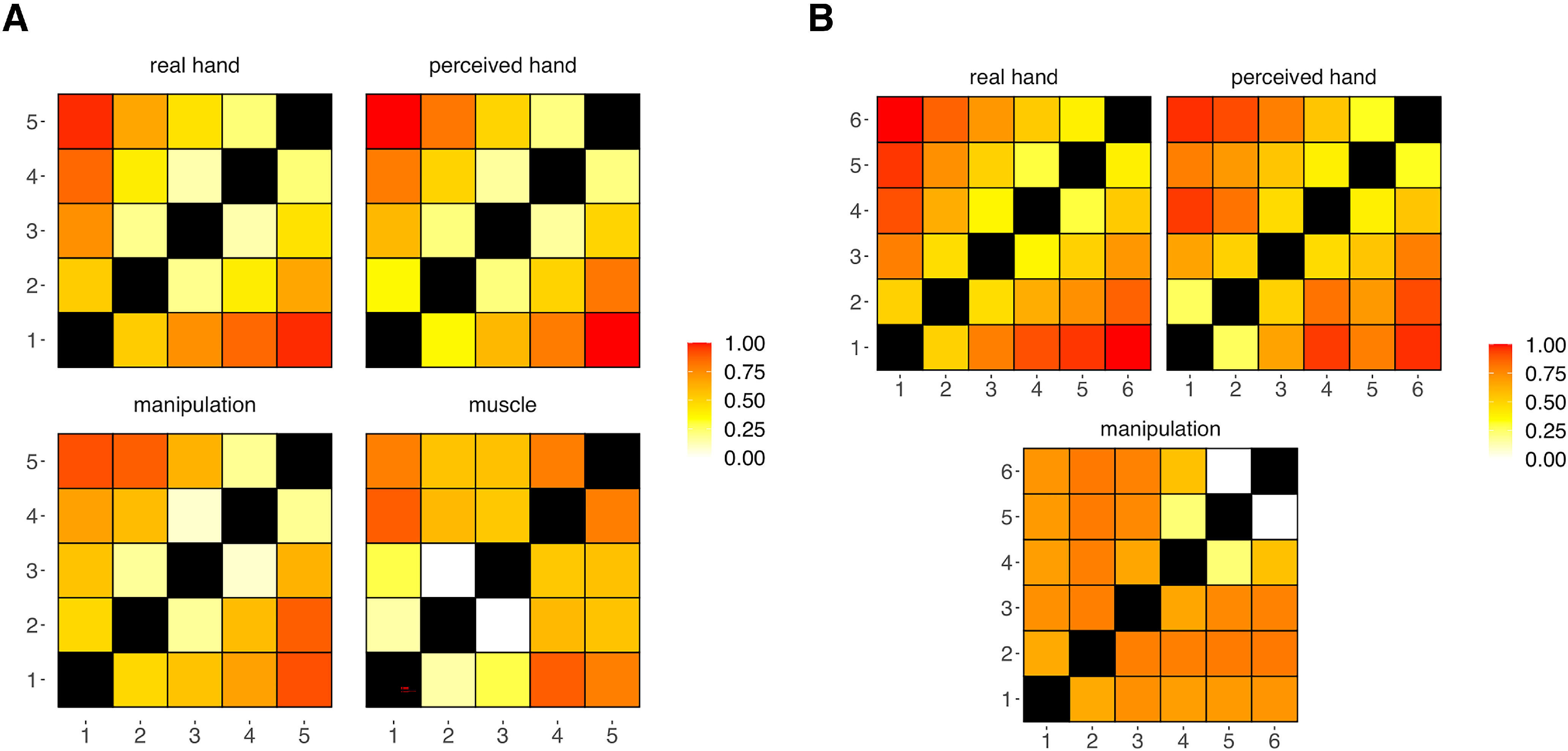
Models tested in 5-fingered subjects (***A***) and in a polydactyly individual (***B***): *real hand* model: relative positions of fingers at rest (i.e., physical hand structure); *perceived hand* model: perceived relative positions of fingers at rest from a hand landmark localization task (i.e., perceived hand structure); *manipulation* model: movement synergies between fingers (finger kinematics) during object manipulation; *muscle* model: muscle synergies as measured through EMG.

We performed representational similarity analysis (RSA) on multivoxel patterns (see RSA) testing four models of FRs: two models of hand structure and two models of hand function (Fig. 3*A*). We use a model of *real hand* structure and a model of *perceived hand* structure obtained from a previous study (Mehring et al., 2019). The *real hand* model was conceived as the physical distances between pairs of fingers (fingertips) at rest. The *perceived hand* model was constructed based on a hand landmark localization task, where participants were blindfolded and were instructed to point toward the cued fingertip with the other hand. From this task, we calculated the perceived positions of the fingertips and their relative distances. Electromyographical recordings of hand muscles and movement kinematics from 6 subjects were collected to compute two models of hand function. A *manipulation* model described the codependencies of finger kinematics during object manipulation. A second model described the cocontraction of hand muscle during single-finger movements (*muscle*). The construction of the *muscle* and the *manipulation* model is described in detail in the following paragraphs.

Six healthy right-handed subjects (2 females, mean age = 31.5, SD 5.2) performed a reach-to-grasp-to-manipulate task. On each trial, participants were asked to reach toward, grasp, lift and manipulate a wooden object placed at ∼40 cm from their body midline. The objects (*n* = 12) differed in shape and size (small cube, medium cube, large cube, small sphere, medium sphere, large sphere, small cone, medium cone, large cone, small cylinder, large cylinder, scissor). Participants were instructed to manipulate each object for ∼2-3 s, rotate it using their fingers, and then place it back on the table before returning to the starting position. Participants began each trial with their palm resting on the table. Each participant performed 10 trials for each object (in a block) for a total of 120 trials. Blocks were fully randomized within subjects. Movement kinematics was recorded using a near-infrared motion capture system (Vicon system) with 12 cameras and a sampling rate of 100 Hz. Participants’ right hands were outfitted with five lightweight retro-reflective hemispheric markers (4 mm in diameter) placed on the tip of each finger. An additional marker (1 mm diameter) was placed on each of the 12 objects. Each trial was inspected offline for correct marker identification, and then run through a low-pass Butterworth filter with a 10 Hz cutoff. Onset and termination of the movement were defined by a velocity threshold (10 mm/s). The *manipulation* model was computed using the absolute velocity measured at the tip of each finger. Data were normalized between 0 and 1. Movement similarity was computed, for each participant and object, as the Euclidean distance between the velocity vector of each pair of fingers. For each participant, Euclidean distances were then averaged over the 12 objects. The *manipulation* model represents Euclidean distances averaged over the six single-subject manipulation models.

The same participants completed a single-finger tapping task with their right hand. Participants were instructed to perform individual finger extension movements at a frequency of1 Hz while keeping the palmar side of their hand on a table. The order in which the fingers had to be moved was fully randomized within subjects. Participants performed 6 trials per finger for a total of 30 trials, and each trial lasted 6 s. Muscle activity was recorded using 13 bipolar Ag/AgCl wireless electrodes (Cometa System). Electrodes were placed on abductor pollicis brevis, second, third, and fourth lumbricalis; abductor digit minimi; first, second, third, and fourth dorsun opponents; wrist extensor radialis; wrist extensor ulnaris; wrist flexor radialis; and wrist flexor ulnaris. The signal from each electrode was sampled at 2 kHz. Electromyography (EMG) signals were then offline downsampled at 1 kHz, rectified and low-pass filtered (fourth-order Butterworth filter, fc = 40 Hz). To determine the similarity of finger movements in the muscle space, we computed, separately for each participant, the Euclidean distance between the trial-averaged EMG activity of each pair of fingers. The *muscle* model represents Euclidean distances averaged over the six single-subject muscle models.

#### RSA

We compared the multivoxel pattern dissimilarity matrices with the four competing models of FR (*real hand*, *perceived hand*, *manipulation*, and *muscle* model). We used non-negative multiple regressions to test the variance explained by each of the four models. We chose to use non-negative multiple regressions to prevent negative coefficients, which are not meaningful in the context of weighted RSA models (Khaligh-Razavi et al., 2017). The regressions were conducted separately on the dissimilarity matrix of each subject, hemisphere, area, and task. The dissimilarity matrices and the models were converted to *z* score before the regressions. Each regression had formula: *D ∼ real hand* *+ perceived hand + manipulation + muscle*, where the dependent variable D is the vectorized top triangle of the dissimilarity matrix and the explanatory variables are the vectors obtained from the top triangle of each model. We then performed a repeated-measures ANOVA on the coefficients assigned to the models by the regressions (2 *model* × 2 *gyrus* × 2 *task*). We conducted *post hoc* analysis using paired *t* tests. In addition, we also report pairwise correlations between the four models were run to assess their reciprocal relationship.

The high correlation between explanatory variables in a multiple linear regression can make the estimation of the coefficients unreliable. Therefore, as a control analysis, we considered each model in isolation, performing simple linear regressions, including each model separately (*D ∼ real hand, D ∼ perceived hand, D ∼ manipulation, and D ∼ muscle*). As for the betas assigned by the multiple regressions, the coefficients assigned to the models by the regressions were compared using a repeated-measures ANOVA (2 *model* × 2 *gyrus* × 2 *task*) and paired *t* tests for *post hoc* analyses. For this analysis, we could also estimate the noise ceiling for model fits (Ejaz et al., 2015). To do this, we run simple linear regressions, including the group average dissimilarity matrix as a “model.” For each individual, we considered the group mean in which this individual was removed. The noise ceiling for each task and gyrus was calculated as the average of the resulting regression coefficients.

#### RSA in a polydactyly subject

We conducted the same analyses in a male polydactyly subject with 6 fully developed fingers on each hand, which allowed us to test our hypothesis in a subject with different hand structure and different hand function and to assess the generalizability of our results across different neuromechanical variants (5-fingered vs 6-fingered individuals). The description of the anatomy, motor and sensory functions, as well as cortical FRs of this polydactyly subject has been previously reported (Mehring et al., 2019). The subject showed overall superior manipulation abilities compared with 5-fingered subjects and had no difficulties in performing finger tapping movements. Also, he did not report any sensory abnormalities or pain associated to the stimulation of the fingers. Similarly to the group of 5-fingered subjects, the polydactyly subject participated in an fMRI experiment with a motor mapping experiment (i.e., tapping task) and a tactile mapping experiment (i.e., tactile stimulation task). The tasks were identical to the ones used for the 5-fingered subjects, except we used blocks of 20 s of stimulation with four repetitions per finger and tactile stimulation was delivered on the palmar side of the hand. All the analyses on the polydactyly subject were conducted in the native space of the subject. Details about MR acquisitions can be found in Mehring et al. (2019). A GLM (GLM1-6F), equivalent to the GLM2 described in GLMs, was conducted in SPM on the preprocessed images in their native space. The model included 6 regressors (one for each of the 6 fingers) and 6 rigid-body motion parameters. In order to perform the multivoxel pattern analysis, we computed a GLM (GLM2-6F) analysis with 24 regressors and 6 rigid body motion regressors per run.

Hand ROI selection was conducted using the GLM1-6F as described in ROI selection. However, while for 5F-subjects we computed a second-level group analysis using subject-level GLM1, for the 6Fsubject we conducted the analysis at the single-subject level.

We computed the cross-validated Mahalanobis distances between activity patterns obtained from the GLM2-6F in each Hand ROI, thus obtaining a 6 × 6 dissimilarity matrix for the motor and a 6 × 6 dissimilarity matrix for the tactile FRs in the precentral and postcentral gyrus (4 in total).

The models of real and perceived hand structure, and object manipulation (Fig. 3*B*) were computed with a procedure similar to the one followed for the 5-fingered subjects (see Models of FRs) and are described in detail in Mehring et al. (2019). The similarity between models was assessed through pairwise correlations between the models. Unfortunately, we could not collect data for the *muscle* model in this subject; therefore, only three models were included in the analysis. The fitting of the models was computed using a non-negative multiple linear regression with formula: *D ∼ real hand* *+ perceived hand + manipulation*, where the dependent variable *D* is the vectorized top triangle of the dissimilarity matrix and the explanatory variables are the vectors obtained from the top triangle of each model.

All statistical analyses were conducted using R (version 3.6.3). Significance level for main and *post hoc* analyses was set to *p* = 0.05. FDR correction was applied for the number of *post hoc* tests computed within each individual analysis.

## Results

Using fMRI, we recorded brain activity during tactile stimulation and movement of each of the 5 fingers on the right and left hands of 25 healthy participants. Fifty hands (25 subjects × 2) were included in the statistical analyses. We studied the degree of convergence or dissociation between FRs recruited by motor or tactile tasks in the precentral and postcentral gyrus. First, we analyzed the morphologic properties (volume of activation and somatotopic ordering) of FRs across cortical areas and tasks. Second, we used the fine-grained cortical activity patterns associated to each finger (Fig. 2*A*) and the connectivity between FRs (Fig. 2*B*) to investigate the representational and anatomic-functional properties of FRs in the precentral and postcentral gyrus as a function of the task, movement, or tactile stimulation of the fingers.

### Volume of activation and somatotopic ordering

**Figure 4.**
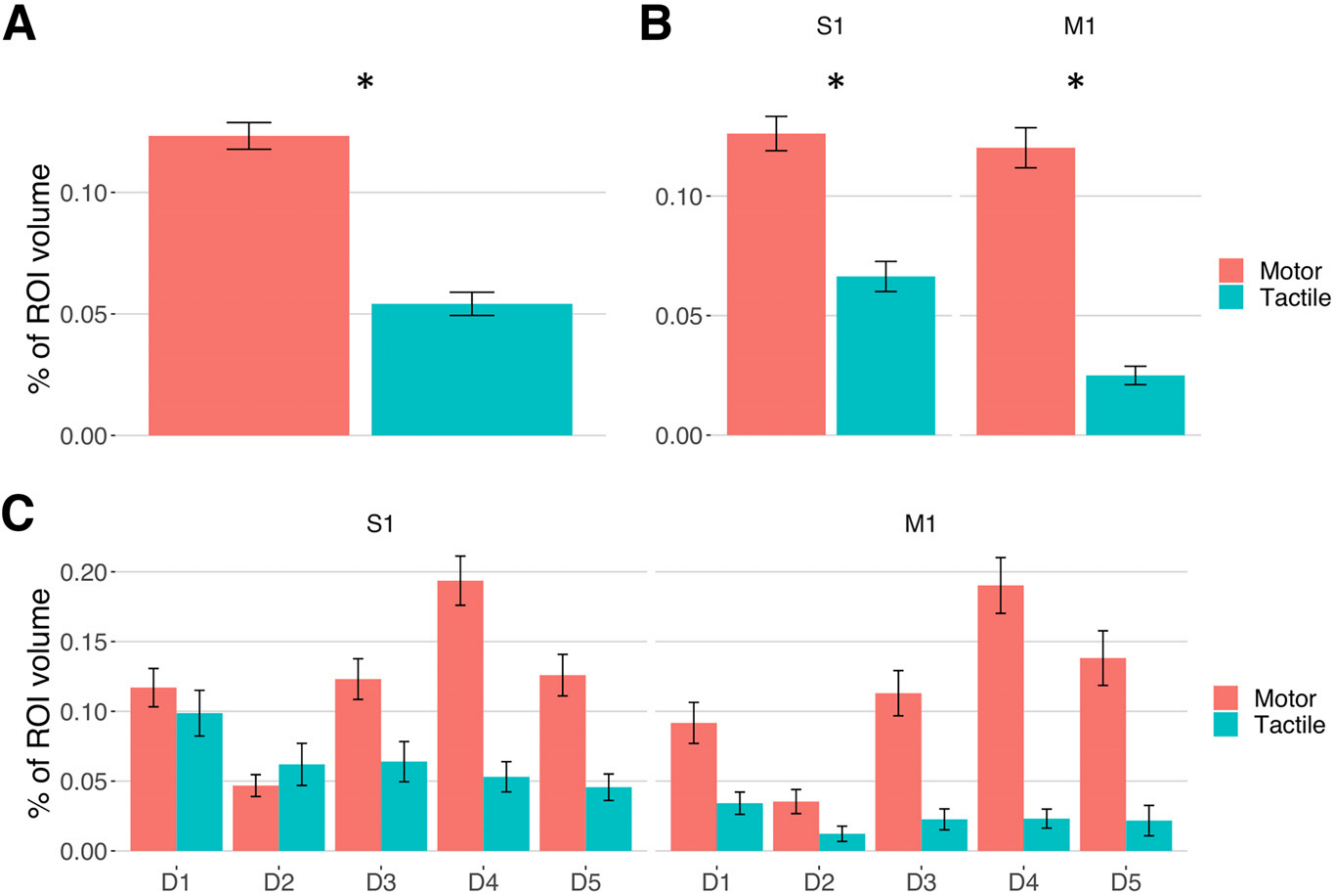
Average volumes of activation of the fingers in the motor and tactile modality in the sensorimotor cortex (***A***) and within the precentral and postcentral gyrus (***B***). ***C***, The volume of activation of each finger during the tactile and motor mapping and their overlap in the precentral and postcentral gyrus. Error bars indicate SEM. *p < 0.05. For relevant comparisons and significant results in ***C***, refer to the text.

**Figure 5.**
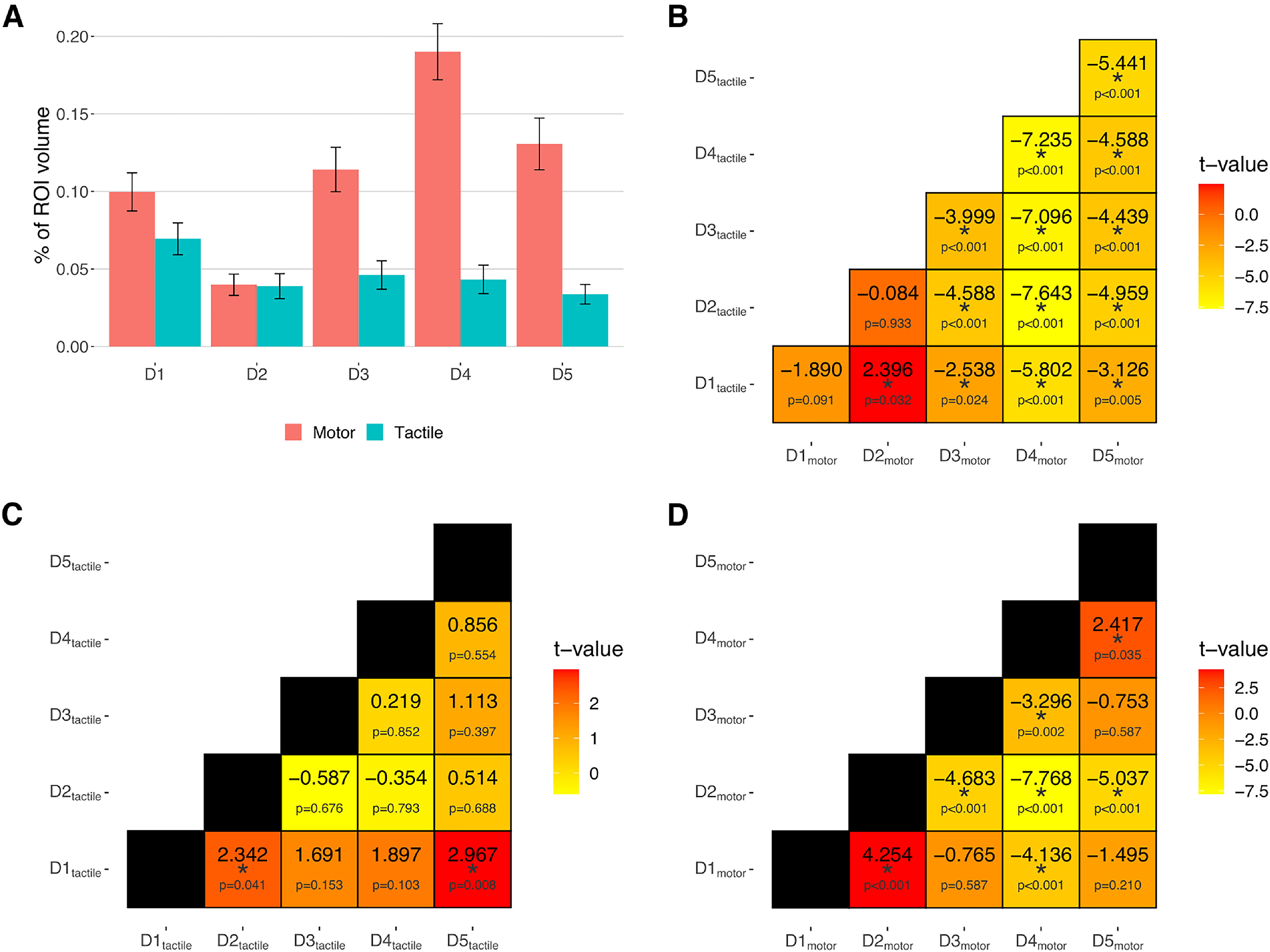
The figures summarize the *post hoc* tests on the interaction effect between *finger* and *modality* found by the repeated-measures ANOVA on the volumes of activation. In the bar plots (***A***), error bars indicate SEM. Matrices represent the *t* and *p* values of the pairwise *t* tests between finger volumes of activation in the tactile versus the motor task (***B***), and during the tactile (***C***) and motor task (***D***). *p < 0.05.

The repeated-measures ANOVA, run on the volumes of the FRs with the factors *finger* (D1-D5), *modality* (tactile and motor), and *gyrus* (precentral and postcentral gyrus), revealed a main effect of *finger* (*F*_(4196)_ = 18.37, *p* < 0.001, η^2^_G_ = 0.0398) and *modality* (*F*_(1,49)_ = 72.15, *p* < 0.001, η^2^_G_ = 0.0974) and significant interactions between *gyrus* and *modality* (*F*_(1,49)_ = 3.96, *p* = 0.047, η^2^_G_ = 0.0057) and between *finger* and *modality* (*F*_(4196)_ = 8.96, *p* < 0.001, η^2^_G_ = 0.0481). *Post hoc* analysis on the main effect of *finger* showed that, considering the average volume in both areas and modalities, the volume of D2 was smaller (t_D2-D1_ = −4.49, *p* < 0.001, Cohen’s *d* = −0.93; t_D2-D3_ = −5.11, *p* < 0.001, Cohen’s *d* = −1.053; t_D2-D4_= −8.46, *p* < 0.001, Cohen’s *d* = −1.729; t_D2-D5_ = −4.87, *p* < 0.001, Cohen’s *d* = −0.994), while the volume of D4 was greater compared with the other fingers (t_D4-D1_ = 4.22, *p* < 0.001, Cohen’s *d* = 0.844; t_D4-D2_ = 8.45, *p* < 0.001, Cohen’s *d* = 1.729; t_D4-D3_ = 3.51, *p* < 0.001, Cohen’s *d* = 0.702; t_D4-D5_ = 2.74, *p* = 0.007, Cohen’s *d* = 0.547). No significant differences were found between the remaining fingers. The overall volume of activation was greater for the motor than the tactile modality (*t* = 5.97, *p* < 0.001, Cohen’s *d *= 1.207) (Fig. 4*A*). Moreover, tactile stimulation induced a significantly larger activation in the postcentral gyrus than in the precentral gyrus (*t* = 3.06, *p* = 0.003, Cohen’s *d* = 0.686) (Fig. 4*B*; for *post hoc* analyses on the interaction between *finger* and *modality*, see Fig. 5).

The cortical magnification of the thumb has been reported since early studies on the human sensorimotor cortex (Penfield and Boldrey, 1937; Woosley et al., 1952) and has been linked with the greater specialization of this finger in sensory and motor functions (Krubitzer et al., 2004; Martuzzi et al., 2014). Thus, we also investigated in detail the difference between the volume of activation of D1 and the other fingers (Fig. 4*C*). The tactile map of D1 in the postcentral gyrus showed a greater volume compared with all the other fingers (t_D1-D2_ = 3.76, *p* < 0.001, Cohen’s *d* = 0.802; t_D1-D3_ = 4.23, *p* < 0.001, Cohen’s *d* = 0.883; t_D1-D4_ = 3.85, *p* < 0.001, Cohen’s *d* = 0.741; t_D1-D5_ = 3.85, *p* < 0.001, Cohen’s *d* = 0.703). No difference between D1 and the other fingers was found for the tactile responses in the precentral gyrus (t_D1-D2_ = 2.26, *p* = 0.051, Cohen’s *d* = 0.715; t_D1-D3_ = 0.4, *p* = 0.706, Cohen’s *d* = 0.163; t_D1-D4_ = 0.62, *p* = 0.561, Cohen’s *d* = 0.254; t_D1-D5_ = 1.06, *p* = 0.368, Cohen’s *d* = 0.528). The motor D1 in the precentral gyrus had a larger activation volume compared with D2 and a smaller activation volume compared with D3, D4, and D5. (t_D1-D2_ = 5.72, *p* < 0.001, Cohen’s *d* = 1.032; t_D1-D3_ = −2.23, *p* = 0.031, Cohen’s *d* = −0.354; t_D1-D4_ = −7.68, *p* < 0.001, Cohen’s *d* = −1.171; t_D1-D5_ = −4.28, *p* < 0.001, Cohen’s *d* = −0.653). In the postcentral gyrus, the motor representation of the thumb was significantly larger than D2 and smaller than D4, while no difference was found with respect to the other fingers (t_D1-D2_ = 6.02, *p* < 0.001, Cohen’s *d* = 1.032; t_D1-D3_ = −0.81, *p* = 0.424, Cohen’s *d* = −0.118; t_D1-D4_ = −5.01, *p* < 0.001, Cohen’s *d* = −0.724; t_D1-D5_ = −0.92, *p* = 0.361, Cohen’s *d* = −0.135).

Concerning the somatotopic ordering, the Bayesian ANOVAs strongly supported the hypothesis of ordering in both the motor and tactile modality in the postcentral gyrus suggesting a lateromedial serial arrangement from D1 to D5. For the tactile FRs in the postcentral gyrus, the ordering hypothesis, H2, obtained a Bayes factor of 13.98, and a posterior probability of 0.933, corresponding to a Bayesian error probability of 0.07. Similar results were found for the motor FRs in the postcentral gyrus with Bayes factor of 8.07, posterior probability of 0.89, and Bayesian error probability of 0.11.

No somatotopic order was found in the precentral gyrus. The Bayesian ANOVA on the motor FRs in the precentral gyrus supported the hypothesis of equivalence of the distances between D1 and the other fingers (D1-D2 ≈ D1-D3 ≈ D1-D4 ≈ D1-D5) with Bayes factor of 7.29, posterior probability of 0.844, and Bayesian error probability of 0.02. Regarding the tactile FRs in the precentral gyrus, the equivalence hypothesis was supported only with a low Bayes factor (1.85) and posterior probability (0.65).

To summarize, we found that the tactile FRs in the postcentral gyrus were macroscopically organized following a clear somatotopic gradient, where the fingers are represented in a sequence from D1 to D5, reflecting their physical spatial arrangement along the hand. No sign of this somatotopic order was found for tactile FRs in the precentral gyrus. Moreover, in line with previous studies (Martuzzi et al., 2014), we found a larger volume of activation of tactile D1 compared with the other fingers in the postcentral gyrus, while the volume of tactile FRs in the precentral gyrus lacked a marked differentiation.

Regarding the motor FRs, a somatotopic order was present in the postcentral gyrus but not in the precentral gyrus. In both areas, we observed a larger volume of activation for the thumb compared with the index finger, while D4 in the postcentral gyrus and D3, D4, and D5 in the precentral gyrus had a larger volume of activation compared with the thumb. We interpret this pattern as an effect of the effort of extending D3, D4, and D5 independently during the task, since these fingers have more reciprocal anatomic constraints.

### PCM

**Figure 6.**
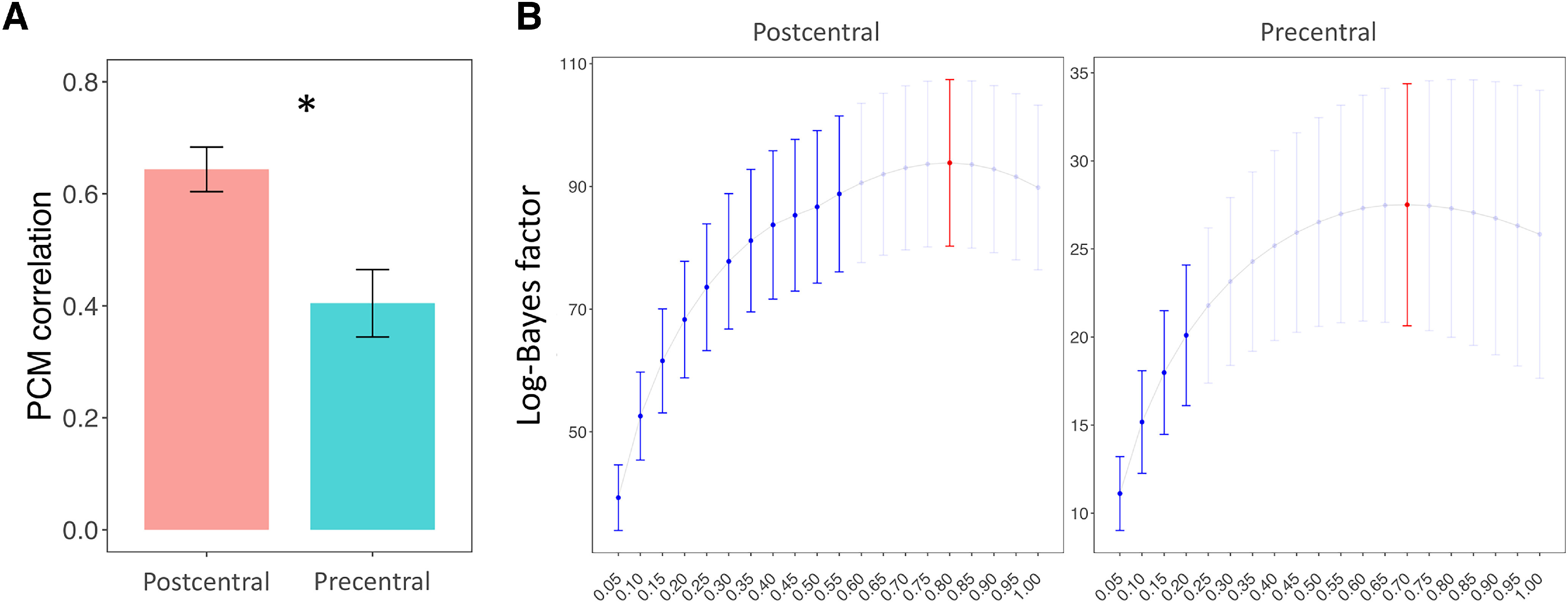
Plots represent the results of the PCM. ***A***, Correlation coefficients estimated using PCM. Error bars indicate SEM. *p < 0.05. ***B***, Comparison between the log-likelihood of the 20 models of correlation between motor and tactile activity patterns, ranging from *r* = 0.05 to *r* = 1, computed using PCM. Error bars indicate SEM. The best fitting models (red) in the postcentral (*r* = 0.8) and in the precentral (*r* = 0.75) were compared with all the other models. Blue represents models that performed significantly differently from the best fitting model.

The correlation between the motor and tactile activity patterns estimated through PCM (Fig. 6*A*) was significantly higher in the postcentral than in the precentral gyrus (*t*_(49)_ = 4.37, *p* < 0.001). Since correlation measures can be influence by differences in the signal-to-noise ratio across different areas and conditions, we used PCM to estimate the true correlation between the patterns by comparing the log-likelihoods of 20 correlation models, equally spaced from *r* = 0.05 to *r* = 1 (Fig. 6*B*). The best fitting model, namely, the model with the highest log-likelihood (peak of the curve in Fig. 6*B*), predicted a correlation of *r* = 0.8 in the postcentral gyrus and slightly lower in the precentral gyrus (*r* = 0.75). We then compared the log-likelihoods of the best fitting models versus all the other models. By comparing the best fitting models with the perfect correlation model (*r* = 1), we did not find any positive evidence that the patterns related to the tactile and motor FRs were not identical in the two areas (postcentral: *t* = 1.59, *p* = 0.119; precentral: *t* = 0.77, *p* = 0.443). The log-likelihoods of the best correlation models were significantly higher than those of the models predicting *r* ≤ 0.55 in the postcentral and *r* ≤ 0.20 in the precentral gyrus. Therefore, we found evidence that the correlation between the patterns related to the tactile stimulation and the movement of the fingers was in the range of [0.55, 1] in the postcentral gyrus and [0.2, 1] in the precentral gyrus.

n conclusion, when correcting for differences in noise and signal strength, PCM found a reasonably high degree of similarity between the encoding structure of motor and tactile FRs in both the postcentral (*r* = 0.80, [0.55, 1]) and the precentral gyrus (*r* = 0.75, [0.2, 1]). However, PCM did not show positive evidence in favor of a different encoding structure of the two modalities.

### Multivoxel pattern analysis

**Figure 7.**
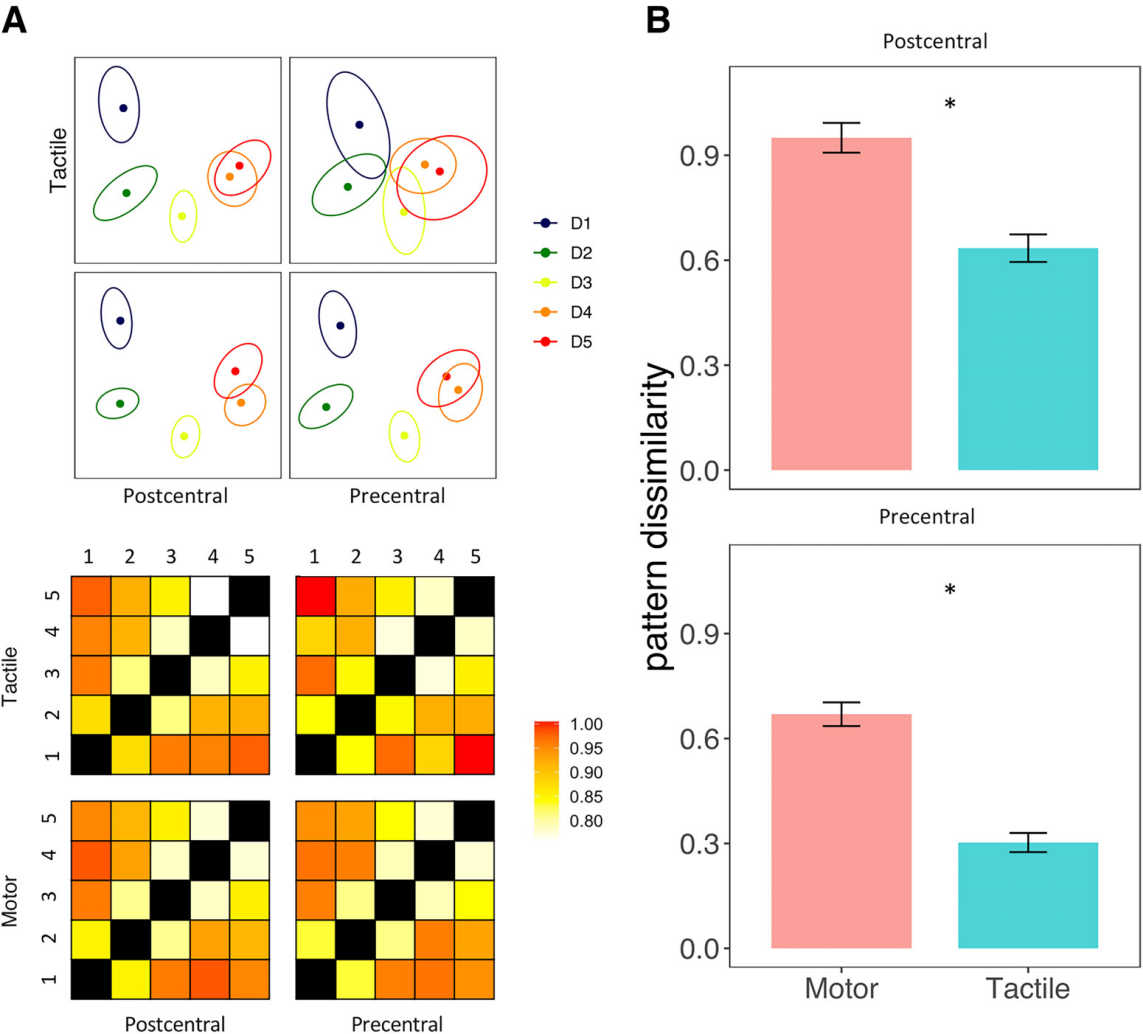
***A***, Four top plots, 2D projections of the multivoxel pattern dissimilarity matrices, which are displayed in the four plots at the bottom of the panel. Error ellipses indicate SEM. ***B***, Mean pattern dissimilarity between FRs within the precentral and postcentral gyrus in the motor and tactile modality. The Mahalanobis distances between patterns are unitless and simply reported in units of pattern dissimilarity. Error bars indicate SEM. *p < 0.05.

We used the multivoxel patterns associated with each finger to analyze each set of FRs separately (motor in the precentral gyrus, tactile in the precentral gyrus, motor in the postcentral gyrus, and tactile in the postcentral gyrus) (Fig. 7*A*).

The repeated-measures ANOVA on the average pattern dissimilarity (Fig. 7*B*) showed a main effect of *task* (*F*_(1,49)_= 159.06, *p* < 0.001, η^2^_G_ = 0.3101) and *gyrus* (*F*_(1,49)_ = 144.36, *p* < 0.001, η^2^_G_ = 0.2664). The discriminability between fingers was higher in the motor than the tactile modality (*t*_(49)_ = 12.61, *p* < 0.001, Cohen’s *d* = 1.783) and higher in the postcentral gyrus than in the precentral gyrus (*t*_(49)_ = 12.01, *p* < 0.001, Cohen’s *d* = 1.7). The largest pattern distances were found between the motor FRs in the postcentral gyrus (0.95 ± 0.3), while the tactile FRs in the precentral gyrus showed the lowest degree of discriminability (0.3 ± 0.19). Despite these differences, the FRs showed the same prototypical arrangement in all conditions and areas reminiscent of the radial arrangement of fingers on the hand (Fig. 7*A*).

**Figure 8.**
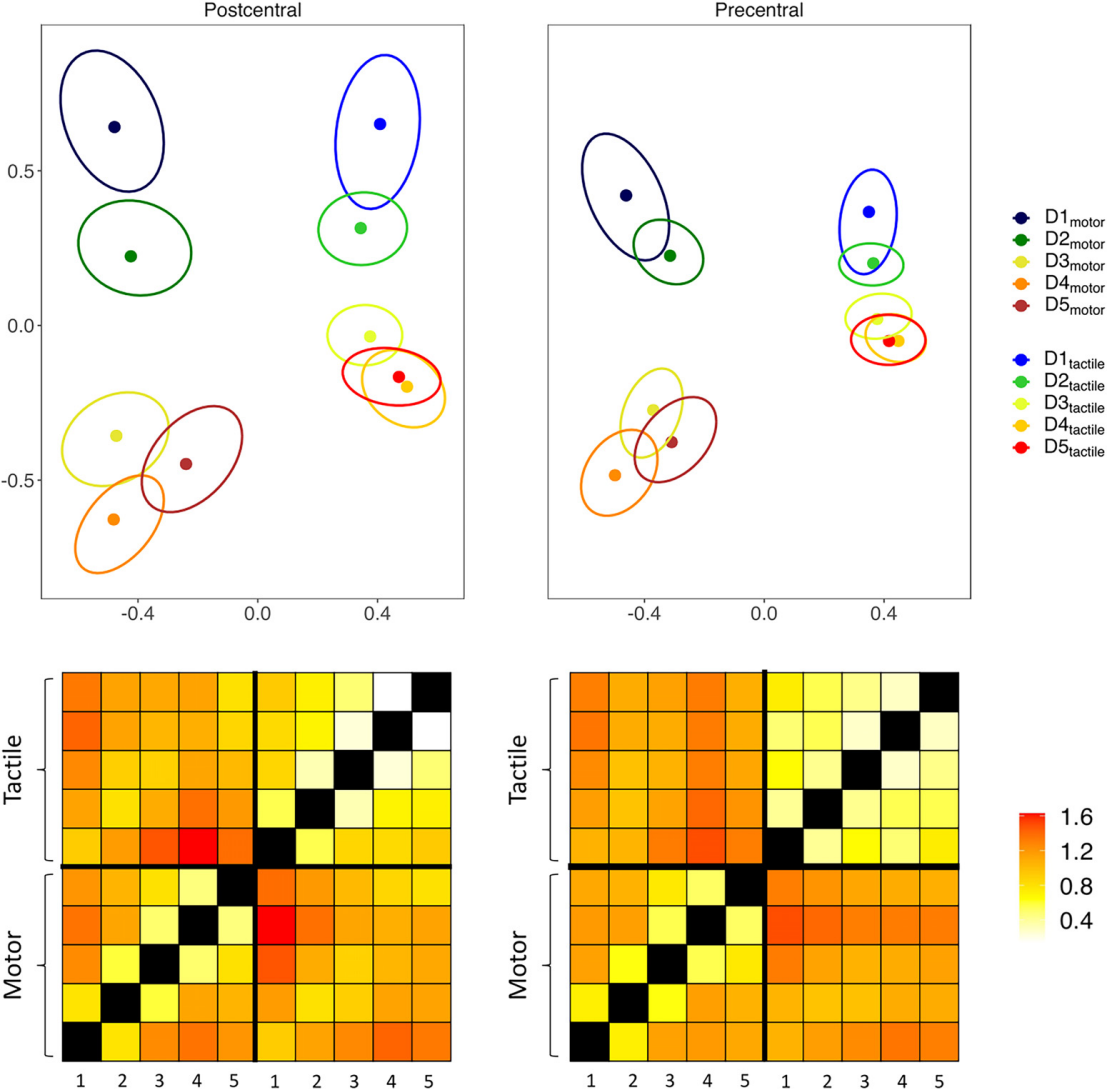
Top plots, 2D projections of the multivoxel pattern dissimilarity matrices, which are displayed at the bottom of the figure. Error ellipses indicate SEM.

**Figure 9.**
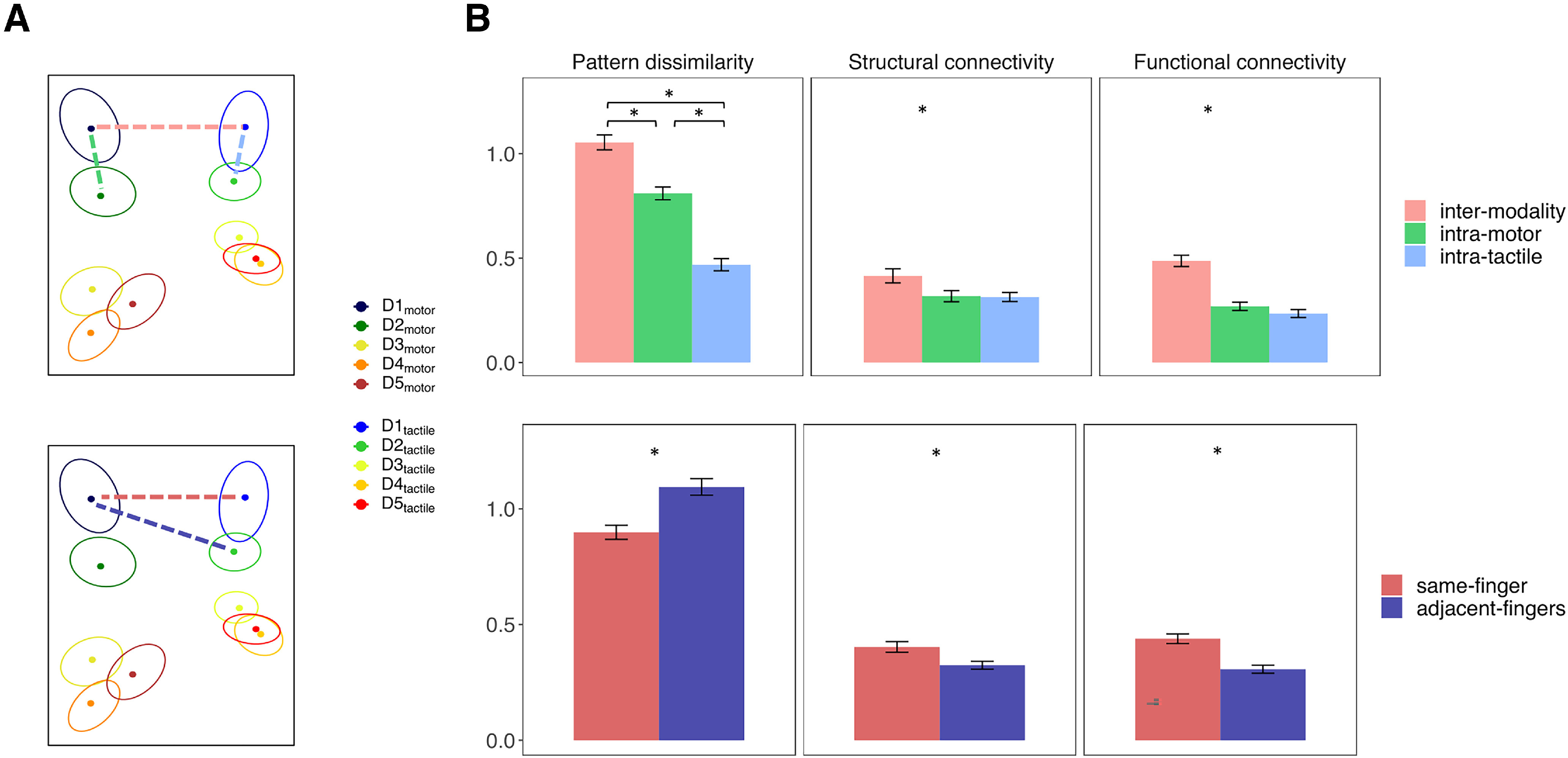
***A***, Graphical example of the measures considered for the analysis of the activity patterns. Top plot, Dotted lines indicate the intermodality (e.g., D1_motor_ – D1_tactile_, pink), intramotor (e.g., D1_motor_ – D2_motor_, green), and intratactile (e.g., D1_tactile_ – D2_tactile_, pale blue) pattern dissimilarities. Bottom plot, Dotted lines indicate the pattern dissimilarity between homologous (e.g., D1_motor_ – D1_tactile_, red) and adjacent FRs across modalities (e.g., D1_motor_ – D2_tactile_, blue). The same definitions apply for the analysis of the functional and structural connectivity patterns. ***B***, Bar plots represent the pattern dissimilarity (unitless), the resting state functional connectivity and the structural connectivity (normalized fiber count) between FRs. Larger pattern dissimilarity indicates greater differences between representations, while greater resting state and structural connectivity indicate stronger anatomic connections and functional interactions. Error bars indicate SEM. *p < 0.05.

We further analyzed the representational geometry of the fingers when considering both modalities together: motor and tactile FRs in the precentral gyrus, motor, and tactile FRs in the postcentral gyrus (Fig. 8) (see the study limitation section for a discussion about the overlap of somatosensory stimulation in the two tasks). If the tactile and motor FRs were encoded separately, a greater dissimilarity between FRs would be expected across modality rather than within modality. We considered the average dissimilarity between the FRs of the same finger across modalities (e.g., D1_motor_ – D1_tactile_) and the average dissimilarity between adjacent fingers within modalities (e.g., D1_motor_ – D2_motor_) (Fig. 9*A*, top row). Repeated-measures ANOVA revealed a main effect of *modality* (*F*_(2,98)_ = 210.52, *p* < 0.001, η^2^_G_ = 0.42). The dissimilarity between fingers across modalities was significantly greater than the dissimilarity between adjacent fingers within the motor (*t*_(49)_ = 11.14, *p* < 0.001, Cohen’s *d* = 1.576) and tactile modality (*t*_(49)_ = 16.54, *p* < 0.001, Cohen’s *d* = 2.34). Motor FRs were also better discriminated overall than tactile FRs (*t*_(49)_ = 12.61, *p* < 0.001, Cohen’s *d* = 1.783) (Fig. 9*B*, bottom row). Finally, we also found a main effect of *gyrus* (*F*_(1,49)_ = 11.92, *p* < 0.001, η^2^_G_ = 0.2015), with greater dissimilarity between fingers in the postcentral than in the precentral gyrus.

We further investigated the dissociation between FRs across the two modalities to detect a possible finger-specific organization across modalities (Fig. 9, bottom row). Thus, we compared the average dissimilarity between the tactile and motor representations of each finger (same-finger, e.g., D1_motor_ – D1_tactile_) with the average dissimilarity across modalities of nonhomologous adjacent fingers (adjacent fingers, e.g., D1_motor_ – D2_tactile_) (Fig. 9*A*, bottom row). We found a main effect of *finger pairs* (*F*_(1,49)_ = 250.43, *p* < 0.001, η^2^_G_ = 0.0872), with greater dissimilarity between different fingers with respect to same-finger (Fig. 9*B*, bottom row) and a main effect of *gyrus* (*F*_(1,49)_ = 33.59, *p* < 0.001, η^2^_G_ = 0.062), with an overall greater discriminability in the postcentral compared with the precentral gyrus.

Thus, on the one hand, we found a greater dissimilarity between the finger-specific cortical patterns across rather than within modality, indicating a dissociation between the encoding of tactile and motor processing in both areas. On the other hand, in both the precentral and postcentral gyrus, we observed a higher similarity between the patterns associated to the movement and the tactile stimulation of the same finger (e.g., D1_motor_ – D1_tactile_) compared with adjacent fingers across modalities (e.g., D1_motor_ – D2_tactile_), further supporting the existence of a finger-specific organization across modalities in both the precentral and postcentral gyrus.

We also found a greater dissimilarity between in the motor than in the tactile modality and in the postcentral than in the precentral gyrus.

### Analysis of the connectivity patterns

We then compared the structural and functional connectivity between FRs within and across modalities (Fig. 9, top row), as well as across modality between FRs of same-finger versus adjacent fingers (Fig. 9, bottom row). To control for the anatomic proximity of the different FRs, we corrected for the Euclidean distances between ROIs.

For both the structural and functional connectivity analyses, the repeated-measures ANOVA showed a main effect of *modality* (structural: *F*_(2,98)_ = 4.26, *p* = 0.029, η^2^_G_ = 0.0293; functional: *F*_(2,98)_ = 67.63, *p* < 0.001, η^2^_G_ = 0.2292) and a main effect of *gyrus* only for functional connectivity (functional *F*_(1,49)_ = 29.96, *p* < 0.001, η^2^_G_ = 0.1239). The connectivity between FRs across modalities was significantly greater than the connectivity within the motor (structural: *t*_(49)_ = 2.22, *p* = 0.047, Cohen’s *d* = 0.32; functional: *t* = 9.04, *p* < 0.001, Cohen’s *d* = 1.278) and tactile (structural: *t*_(49)_ = 2.8, *p* = 0.022, Cohen’s *d* = 0.404; functional: *t*_(49)_ = 9.84, *p* < 0.001, Cohen’s *d* = 1.392) modalities. The functional connectivity was also significantly higher in the postcentral compared with the precentral gyrus (*t*_(49)_ = 5.47, *p* < 0.001, Cohen’s *d* = 0.774).

Regarding the existence of specific anatomic and functional connections across modality between FRs of the same-finger compared with adjacent-finger, we found a main effect of *finger pairs* for both types of connectivity (structural: *F*_(1,49)_ = 15.26, *p* < 0.001, η^2^_G_ = 0.04; functional: *F*_(1,49)_ = 47.18, *p* < 0.001, η^2^_G_ = 0.1211) and a main effect of *gyrus* for the functional connectivity (functional: *F*_(1,49)_ = 16.64, *p* < 0.001, η^2^_G_ = 0.1198). The connectivity between the representations of the same finger across modality was significantly greater than the connectivity between adjacent fingers (structural: *t*_(49)_ = 3.9, *p* < 0.001, Cohen’s *d* = 0.564; functional: *t*_(49)_ = 6.87, *p* < 0.001, Cohen’s *d* = 0.971). Again, the functional connectivity across modality was higher in the postcentral than in the precentral gyrus (*t*_(49)_ = 4.08, *p* < 0.001, Cohen’s *d* = 0.577).

To summarize, consistently with the representational geometry of the fingers, we found a greater structural and resting state functional connectivity between the representations of the same finger compared with adjacent fingers across modalities (e.g., D1_motor_ – D1_tactile_ vs D1_motor_ – D2_tactile_), suggesting that the tactile and motor representations of each finger form a distinct anatomic-functional unit. We also found a prevalence of anatomic connections and functional interactions across rather than within modalities (e.g., D1_motor_ – D1_tactile_ vs D1_motor_ – D2_motor_ and D1_tactile_ – D2_tactile_), while the analysis of the cortical patterns revealed an opposite relationship (greater dissimilarity within rather than between modalities, see previous paragraph).

The mapping of the U-fibers connecting nearby cortical areas at the gray-white matter boundary is particularly difficult at the voxel resolution used for this study. Therefore, our results on structural connectivity should be taken with caution. It is worth noticing that the resolution of fiber directions is normally improved using a high number of gradient directions (in this case, *n* = 137). Moreover, the results on structural connectivity are coherent with the results obtained by using different techniques (functional connectivity and multivoxel pattern analysis).

Together, these results suggest that individual motor and tactile FRs coexist within both the precentral and postcentral gyrus (as shown by the cortical patterns) and can be conceived as part of a local network, in which denser anatomic connections and stronger functional interactions favor the coordinated processing of information between movement and associated somatosensory stimulation of the same finger, more than between adjacent fingers.

### RSA

Using dissimilarity analysis, we investigated the representational geometry of FRs to determine the hand features, as described by competing models of hand representations, that are most likely encoded in the precentral and postcentral gyrus. To achieve this, we searched for the model of hand structure and function that best described the pattern of cortical activity evoked by the motor and the tactile task across the central sulcus. We computed dissimilarity matrices based on multivoxel patterns and compared these matrices with four possible models of FRs. In particular, two models described hand structure (*real hand* and *perceived hand* structure) and two models were related to hand function (*muscle* and *manipulation*) (for a full description of the models, see Models of FRs; Fig. 3*A*).

**Figure 10.**
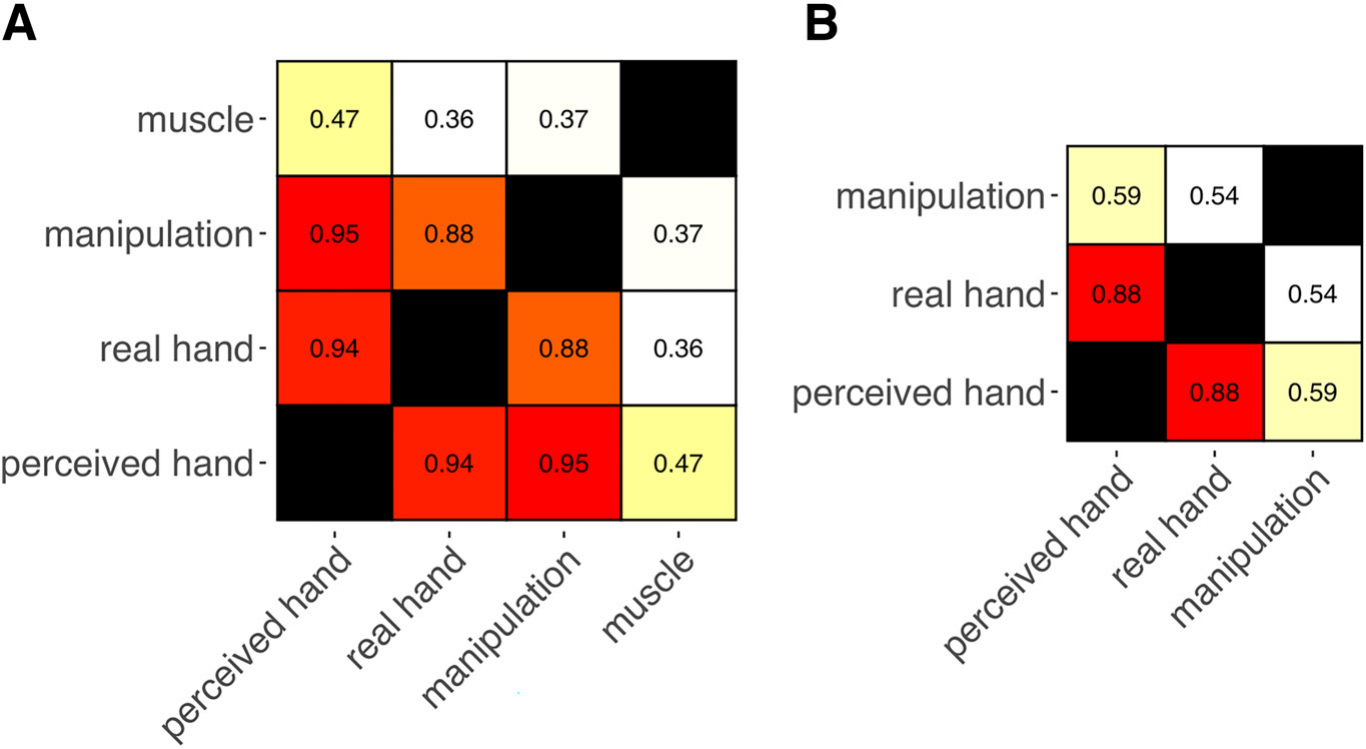
Pairwise correlations between models in 5-fingered subjects (***A***) and in a polydactyly individual (***B***).

Given the fixed anatomic bounds between the fingers, hand structure and function are expected to be to some extent interdependent. Looking at the pairwise correlations between the models (Fig. 10*A*), the *muscle* model showed the lowest degree of similarity with the other models (mean *r* = 0.4). As might be expected, the perceived and the real hand structures were strongly correlated (*r* = 0.94). However, the *perceived hand* model and the *manipulation* model showed the highest degree of similarity (*r* = 0.95), while the correlation between the *real hand* model and the *manipulation* model was lower (*r* = 0.88). Thus, *the perceived hand* model seems to combine features associated with both hand anatomy and function.

**Figure 11.**
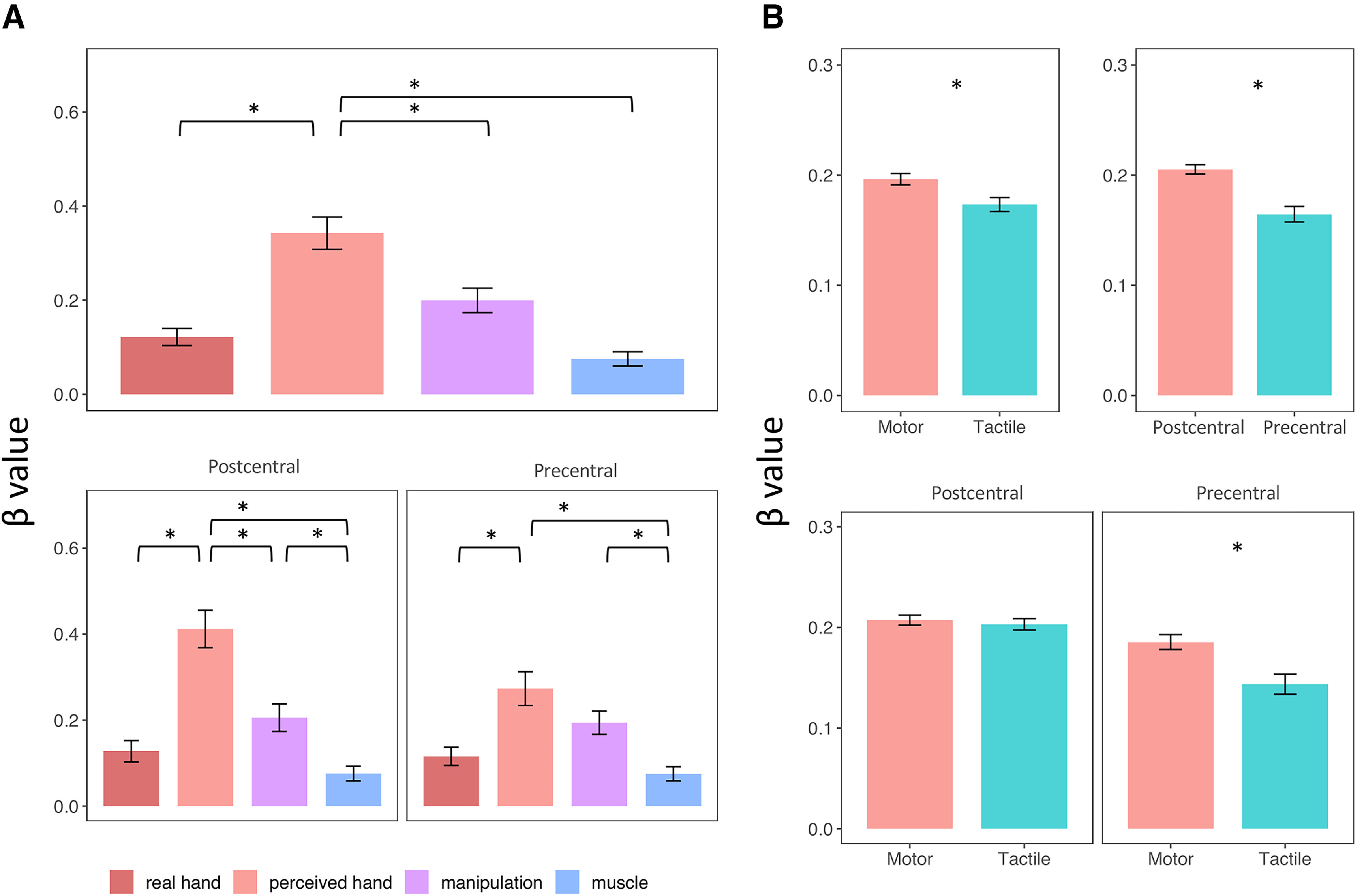
Comparison between the coefficients (β values) assigned to the four models by the non-negative multiple linear regressions on dissimilarity matrices. Plots represent the significant main effects and interactions found by the repeated-measures ANOVA on β values. ***A***, Main effect of *model* (top plot) and interaction between *model* and *gyrus*. ***B***, Main effect of *task* and *gyrus* (top plots) and interaction between these two factors (bottom plots). Error bars indicate SEM. *p < 0.05.

We used non-negative multiple linear regression on the pattern dissimilarity matrices to assess the amount of variance explained by each model (Fig. 11; Table 1). We submitted the coefficients (independently estimated for each subject, hemisphere, task, and cortical area) to a repeated-measures ANOVA (four *model* × 2 *gyrus* × 2 *task*). We found main effects of *model* (*F*_(3,147)_ = 17.49, *p* < 0.001, η^2^_G_ = 0.1352), *task* (*F*_(1,49)_ = 9.64, *p* = 0.003, η^2^_G_ = 0.002), and *gyrus* (*F*_(1,49)_= 29.09, *p* < 0.001, η^2^_G_ = 0.0062) and a significant interaction between *model* and *gyrus* (*F*_(3,147)_ = 3.43, *p* = 0.019, η^2^_G_ = 0.0121) and *gyrus* and *task* (*F*_(1,49)_ = 15.48, *p* < 0.001, η^2^_G_ = 0.0013).

**Table 1.** Regression coefficients assigned by the non-negative multiple linear regressions*^a^*

Model	Gyrus	Task	SD	Mean
Main effect of model			
Manipulation			0.183	0.2
Muscle			0.106	0.007
Perceived hand			0.244	0.342
Real hand			0.129	0.122
Main effect of gyrus
	Postcentral		0.031	0.205
	Precentral		0.049	0.164
Main effect of task
	Motor		0.037	0.196
	Tactile		0.044	0.173
Model × gyrus interaction
Manipulation	Postcentral		0.226	0.206
Manipulation	Precentral		0.192	0.194
Muscle	Postcentral		0.121	0.076
Muscle	Precentral		0.117	0.075
Perceived hand	Postcentral		0.308	0.412
Perceived hand	Precentral		0.279	0.273
Real hand	Postcentral		0.175	0.128
Real hand	Precentral		0.149	0.116
Gyrus × task interaction
	Postcentral	Motor	0.035	0.207
	Postcentral	Tactile	0.04	0.203
	Precentral	Motor	0.052	0.185
	Precentral	Tactile	0.071	0.143

*^a^*Data are split according to the significant main effect and interactions, corresponding to the plots in Figure 11.

*Post hoc* analysis on the main effect of *model* showed that the *perceived* model was overall better than the *real hand* (*t*_(49)_ = 5.34, *p* < 0.001, Cohen’s *d* = 0.756), *manipulation* (*t*_(49)_ = 2.57, *p* = 0.013, Cohen’s *d* = 0.364), and *muscle* model (*t*_(49)_ = 6.19, *p* < 0.001, Cohen’s *d* = 0.876). The manipulation model was better than both the real (*t*_(49)_ = 2.19, *p* = 0.04, Cohen’s *d* = 0.31) and the muscle model (*t*_(49)_ = 4.07, *p* < 0.001, Cohen’s *d* = 0.577). The interaction between *model* and *gyrus* was mainly explained by the fact that the *perceived hand* model was better than the *real hand* (*t*_(49)_ = 4.96, *p* < 0.001, Cohen’s *d* = 0.702), *manipulation* (*t*_(49)_ = 2.97, *p* = 0.005, Cohen’s *d* = 0.421), and *muscle* model (*t*_(49)_ = 6.38, *p* < 0.001, Cohen’s *d* = 0.903) in the postcentral gyrus, while it was significantly better than the *real hand* (*t*_(49)_ = 3.33, *p* = 0.002, Cohen’s *d* = 0.471) and the *muscle* model only (*t*_(49)_ = 4.28, *p* < 0.001, Cohen’s *d* = 0.605) in the precentral gyrus. (Fig. 11*A*).

Regarding the main effects of *task* and *gyrus*, *post hoc* analysis revealed an overall better fitting of the motor compared with the tactile modality (*t*_(49)_ = 3.10, *p* = 0.003, Cohen’s *d* = 0.439) and within the postcentral compared with the precentral gyrus (*t*_(49)_ = 5.39, *p* < 0.001, Cohen’s *d* = 0.763). The interaction between these two factors is because of the lower coefficients assigned to the models in the tactile modality in the precentral gyrus (*t*_(49)_ = −3.86, *p* < 0.001, Cohen’s *d* = −0.547), while the two modalities are comparable in the postcentral gyrus (*t*_(49)_ = 0.66, *p* = 0.507, Cohen’s *d* = 0.095) (Fig. 11*B*).

**Figure 12.**
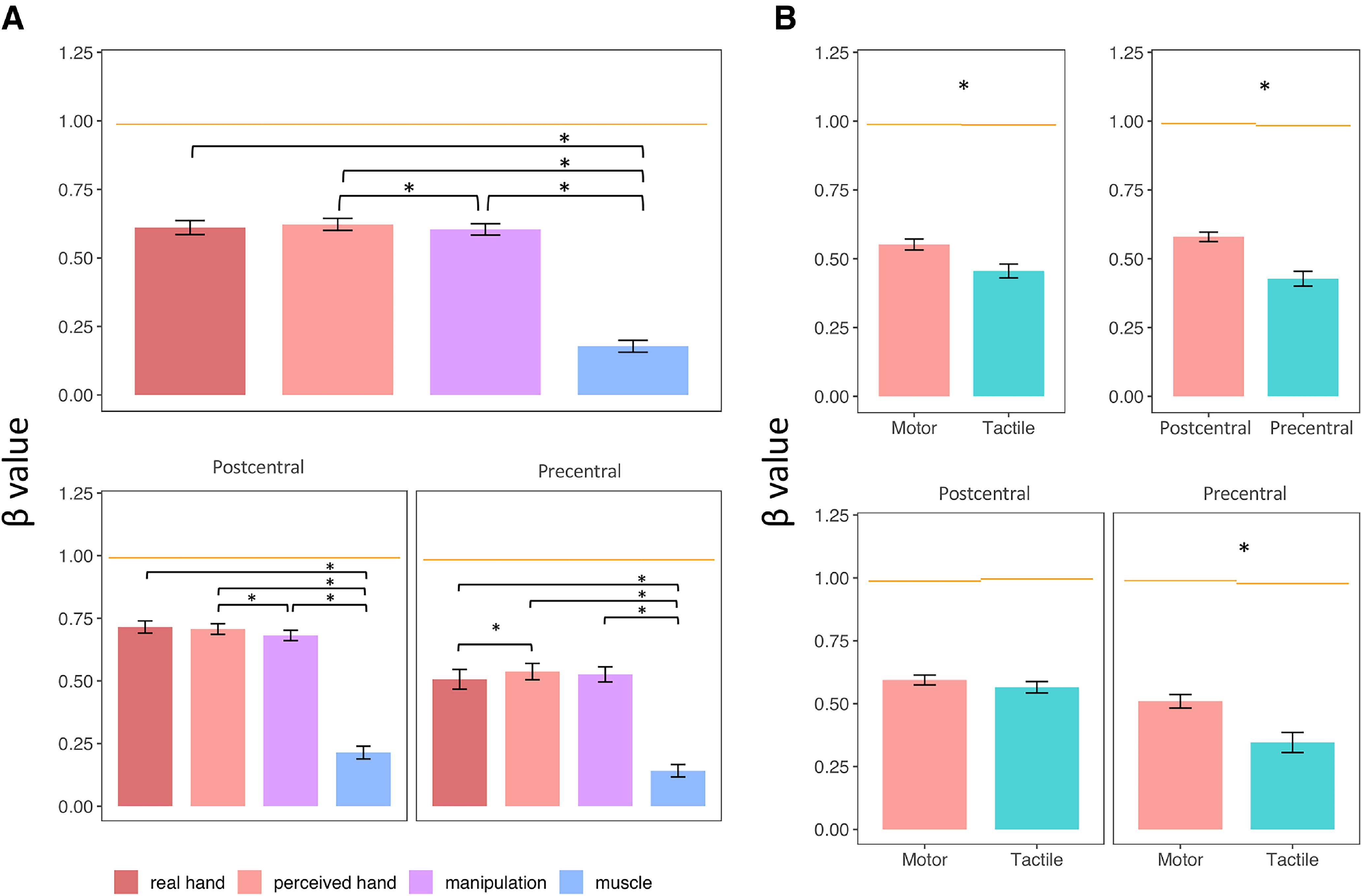
Graphs represent the comparison between the coefficients (β values) assigned to each of the four models by the simple linear regressions. ***A***, Main effect of model (top plot) and interaction between model and gyrus. ***B***, Main effect of taskÚnd gyrus (top plots) and interaction between these two factors (bottom plots). Error bars indicate SEM. *p < 0.05. Horizontal bars represent the noise-ceiling (i.e., the coefficient assigned to the best-possible model).

The collinearity between the models could make the estimate of the regression coefficients in the multiple regressions unreliable. To check for these effects, we also run simple linear regressions including each model separately. Given the high correlation between the *real hand*, *perceived hand*, and *manipulation* model, it was expected that at least some of the differences between these models would be reduced, because they would be ‘hidden’ by the collinearity between the models. However, also in this analysis, we found the same pattern observed when comparing the four models using multiple linear regressions (Fig. 12; Table 2): the *muscle* model performed significantly worse than all the other models; more importantly, the relative advantage of the *perceived hand* model versus all the other models was also qualitatively observed in this analysis.

**Table 2.** Regression coefficients assigned by the independent simple regressions*^a^*

Model	Gyrus	Task	SD	Mean
Main effect of model			
Real hand			0.18	0.61
Perceived hand			0.154	0.622
Manipulation			0.148	0.604
Muscle			0.153	0.178
Main effect of gyrus
	Postcentral		0.122	0.58
	Precentral		0.189	0.428
Main effect of task
		Motor	0.141	0.552
		Tactile	0.178	0.456
Model × gyrus interaction
Real hand	Postcentral		0.173	0.715
Real hand	Precentral		0.279	0.506
Perceived hand	Postcentral		0.15	0.71
Perceived hand	Precentral		0.23	0.54
Manipulation	Postcentral		0.146	0.682
Manipulation	Precentral		0.213	0.526
Muscle	Postcentral		0.18	0.214
Muscle	Precentral		0.175	0.141
Gyrus × task interaction
	Postcentral	Motor	0.14	0.594
	Postcentral	Tactile	0.162	0.565
	Precentral	Motor	0.191	0.51
	Precentral	Tactile	0.281	0.346

*^a^*Data are split according to the significant main effect and interactions, corresponding to the plots in Figure 12.

In detail, ANOVA revealed a significant main effect of *model* (*F*_(3147)_ = 183.99, *p* < 0.001, η^2^_G_ = 0.3662), *gyrus* (*F*_(1,49)_ = 29.53, *p* < 0.001, η^2^_G_ = 0.086), and *task* (*F*_(1,49)_ = 11.31, *p* < 0.001, η^2^_G_ = 0.0363). In a *post hoc* analysis on the main effect of *model* (Fig. 12*A*, top plot), the *perceived hand* model performed better than the *manipulation* model (*t*_(49)_ = 2.37, *p* = 0.033, Cohen’s *d* = 0.335), while the *muscle* model performed significantly worse than all the other models (*real hand* vs *muscle*: *t*_(49)_ = 13.19, *p* < 0.001, Cohen’s *d* = 1.866; *perceived hand* vs *muscle*: *t*_(49)_ = 15.87, *p* < 0.001, Cohen’s *d* = 2.244; *manipulation* vs *muscle*: *t*_(49)_ =14.73, *p* < 0.001, Cohen’s *d* = 2.0283). The fitting of all the models was better in the postcentral gyrus than in the precentral gyrus (*t*_(49)_ = 5.43, *p* < 0.001, Cohen’s *d* = 0.768) and during the motor compared with the tactile mapping (*t*_(49)_ = 3.36, *p* = 0.001, Cohen’s *d* = 0.476) (Fig. 12*B*, top plots). We also found a significant interaction between *model* and *gyrus* (Fig. 12*A*, bottom plots) (*F*_(3147)_ = 7.48, *p* = 0.001, η^2^_G_ = 0.001), because of a better fitting performance of the *perceived hand* model compared with the *real hand* model in the precentral gyrus (*t*_(49)_ = 2.48, *p* = 0.025, Cohen’s *d* = 0.351), but not in the postcentral gyrus (*t*_(49)_ = 0.67, *p* = 0.51, Cohen’s *d* = 0.095). The muscle model performed worse than all the other models both in the postcentral gyrus (*real hand* vs *muscle*: *t*_(49)_ = 14.25, *p* < 0.001, Cohen’s *d* = 2.016; *perceived hand* vs *muscle*: *t*_(49)_ = 16.27, *p* < 0.001, Cohen’s *d* = 2.301; *manipulation* vs *muscle*: *t*_(49)_ = 14.31, *p* < 0.001, Cohen’s *d* = 2.024) and in the precentral gyrus (*real hand* vs *muscle*: *t*_(49)_ = 8.26, *p* < 0.001, Cohen’s *d* = 1.168; *perceived hand* vs *muscle*: *t*_(49)_ = 11.05, *p* < 0.001, Cohen’s *d* = 1.562; *manipulation* vs *muscle*: *t*_(49)_ = 11.09, *p* < 0.001, Cohen’s *d* = 1.569). Finally, a significant interaction between *task* and *gyrus* was found (Fig. 12*B*, bottom plots) (*F*_(1,49)_ = 12.24, *p* < 0.001, η^2^_G_ = 0.0183). Indeed, the fitting of the activity patterns during finger movements and tactile stimulation was comparable in the postcentral gyrus (*t*_(49)_ = 1.13, *p* = 0.263, Cohen’s *d* = 0.16), while it was significantly deteriorated in the precentral gyrus during the tactile stimulation (*t*_(49)_ = −3.9, *p* < 0.001, Cohen’s *d* = 0.554).

Together, these analyses show that the model of *perceived hand* structure best describes the representational geometry of the tactile and motor FRs. The *perceived hand* model is overall better than the other candidate models across the sensorimotor cortex, especially in the precentral gyrus.

### RSA in a polydactyly subject

We then asked whether the features encoded by FRs for both modalities and both gyri depend on the specific neuromechanical properties of the hand. To do so, we studied a polydactyly individual with 6 fully developed and functional fingers, that, in previous study, showed enhanced motor abilities than 5-fingered individuals (Mehring et al., 2019). Thus, in this particular polydactyly individual, the anatomic and functional properties of the hand, as well as their relationship, are very different compared with 5-fingered subjects. Accordingly, we observed that the correlation between both models of hand structure and the model of hand function was much lower in this case than in 5-fingered subjects (*perceived hand* – *manipulation*: *r* = 0.59; *real hand* – *manipulation*: *r* = 0.54) (Fig. 10*B*).

**Figure 13.**
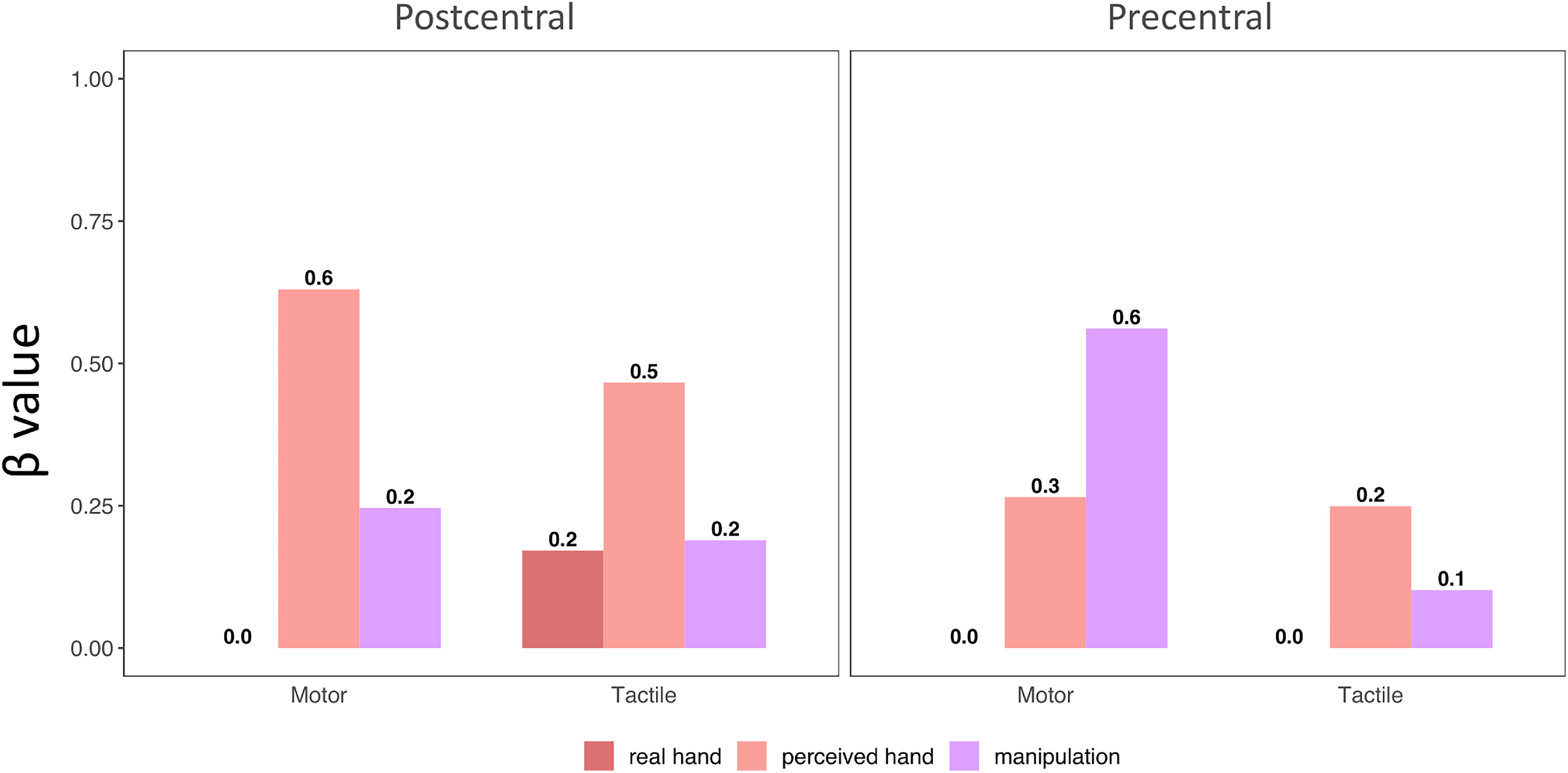
The coefficients assigned to the three models by the non-negative multiple regression on the activity patterns associated to the movement and the tactile stimulation of the fingers, in the precentral and postcentral gyrus of a polydactyly subject with 6 fingers.

As in the previous analysis, we computed the representational geometry of FRs in the precentral and postcentral gyrus for both motor and tactile modalities, and then we compared it through a non-negative multiple regression with three models of hand representation: (1) real hand structure, (2) perceived hand structure, and (3) object manipulation. The *manipulation* model showed the best fitting for the motor FRs in the precentral gyrus, while the *perceived hand* model better described the representational geometry of the tactile FRs in the precentral gyrus and of the FRs in both modalities in the postcentral gyrus (Fig. 13).

Thus, despite the unique features of the 6-fingered hand, the hand structure perceived by this polydactyly subject could still describe better than the *real hand* and the *manipulation* models the FRs for both modalities in the postcentral gyrus and in the tactile modality in the precentral gyrus. However, a dissociation between modalities could be seen in the precentral gyrus, where the manipulation model better fitted the representational geometry of the motor FRs. Although we note that these results are based on single-case observations, and thus should be interpreted with caution, task-dependent encoding of hand features seem to emerge when the anatomic and functional properties of the hand tend to diverge.

## Discussion

In this study, we investigated the dissociation between the processing of movement and isolated tactile stimulation in the precentral and postcentral gyrus using the fingers as study model. In 25 5-fingered participants and in a congenital polydactyl individual, we mapped motor and tactile FRs in the precentral and postcentral gyrus via two independent tasks: active movements and passive tactile stimulation of the fingers. We analyzed FRs across tasks and gyri in terms of activation volume, somatotopic ordering, pattern dissimilarity, and connectivity (structural and functional). Finally, we conducted an RSA to test which hand features are most likely encoded by FRs as a function of task and gyrus.

Both movement and tactile stimulation of the fingers evoked well-defined, finger-specific, and somatotopically organized activations in the postcentral gyrus, in line with previous fMRI studies (Martuzzi et al., 2014; Ejaz et al., 2015; Kolasinski et al., 2016a, 2016b; Berlot et al., 2019; Sanders et al., 2019). Activity elicited by the movement of each finger revealed a lack of somatotopic organization in the precentral gyrus, where motor representations are fragmented and spatially overlapping (Schieber and Hibbard, 1993; Indovina and Sanes, 2001; Olman et al., 2012; Siero et al., 2014; Schellekens et al., 2018). This finding does not fully correspond to the results of Huber et al. (2020), which report multiple somatotopical maps of motor FRs in the precentral gyrus. Future studies with a similar design, but combining higher resolution, signal-to-noise ratio and dedicated sequences (e.g., VASO), will be needed to elucidate this point. However, in the present study, the motor representation of each individual finger could be still detected as a unique and distinct pattern of activity.

Regarding somatosensory representations in the precentral gyrus, we found that the tactile FRs in this area were not somatotopically organized and their relative size did not reveal a marked differentiation between the fingers at a macroscopic level. Moreover, comparing the four sets of FRs (motor in the precentral gyrus, tactile in the precentral gyrus, motor in the postcentral gyrus, and tactile in the postcentral gyrus), we found that the tactile FRs had the lowest level of discriminability (i.e., they were the less differentiated). Yet, the representational geometry of tactile FRs in the precentral gyrus showed a radial arrangement reminiscent of the physical arrangement of the fingers on the hand (Fig. 4*A*), in agreement with previous reports on tactile FRs in the postcentral gyrus (Akselrod et al., 2021) and motor FRs (Ejaz et al., 2015), which is an indication of the existence of differentiated tactile representations of the fingers in the precentral gyrus. These results resonate with data from monkey experiments, which described neurons with large receptive field, usually covering more than one finger or even the entire hand, responding to the tactile stimulation of the hand in the precentral gyrus (Lemon, 1981).

When considering both modalities together (motor and tactile FRs in the precentral gyrus, motor and tactile FRs in the postcentral gyrus), we found a finger-specific cross-modal organization in both the precentral and postcentral gyrus. A similar relationship was found in the structural and functional connectivity patterns between fingers, further supporting the existence of supra-modal FRs in the sensory-motor cortices. The consistency of these tactile–motor interactions in both the precentral and postcentral gyrus indicates that the coupling between homologous FRs across different modalities, mediated by their anatomic and functional connections, might be a major criterion for the organization of the hand area. To gather deeper insight about the function of this organization, we then asked what the content of such somatosensory and motor representations is.

The results of the PCM suggested that the encoding structure of the two modalities might be very similar, if not identical. Indeed, comparing models based on the anatomic (real and perceived hand structure) and functional (muscle synergies and finger kinematics) properties of the hand, we found that the perceived structure of the hand best described the representational geometry of the FRs, regardless of the recruited modality (movement or tactile stimulation of the fingers). Some systematic distortions in hand perception reported by behavioral studies (Longo and Haggard, 2010; Sadibolova et al., 2018) have been put in relationship with early steps in the processing of the somatosensory information. Consistently with this view, our results point to a direct link between the perception of the fingers and their neural representations in the sensorimotor cortex. However, unlike the hand anatomy or models based on the co-occurrence of finger movements, the perceived hand model has no obvious relationship with the physical or functional properties of the hand. Therefore, the reason why the cortical representation of the fingers might be structured according to this particular representational model of perceived hand structure is not straightforward. One way to interpret this result is suggested by the relationship between the different representational models. Indeed, we found that the perceived hand structure was more similar to both the actual hand anatomy and function than these latter two were to each other. Moreover, the perceived hand model seems to describe well the encoding of both sensory and motor information related to the fingers in the precentral and postcentral gyrus. Thus, it can be hypothesized that the representation of the fingers in different areas and modalities might tend to converge toward a common representational model, captured by the perceived hand model, to simplify the neural architecture and facilitate sensorimotor integration processes. This compromise might be advantageous in reducing neuro-computational costs, despite introducing slight perceptual distortions, namely, a perceived hand deviating from the real hand structure.

The result of this adapting process might depend on the neuromechanical properties of the standard hand anatomy. In other words, natural (Mehring et al., 2019) or artificial deviation (Kieliba et al., 2021) from the normal relationship between hand structure and function may impact the way different neural representations of the fingers are internally organized and relate to each other. To approach this question, we considered the case of a polydactyly individual with one anatomically complete and functionally independent supernumerary finger. Although this is a single case, it provides us with the rare opportunity to investigate how the neuromechanical properties of the hand, and the encoding strategy adopted by the sensorimotor cortex, might be reciprocally affected. This individual was able to use the additional finger to perform more complex manipulations than 5-fingered individuals (Mehring et al., 2019). Interestingly, because of the exceptional hand anatomy and the peculiar use of the supernumerary finger, real and perceived hand structure correlated much less with hand function in this individual than in 5-fingered participants.

In the 6-fingered individual, we found that the perceived structure of the hand was the best model for both the motor and tactile FRs in the postcentral gyrus and for tactile FRs in the precentral gyrus, confirming a general tendency across the sensorimotor cortex toward a common representational scheme, independently of the specific hand anatomy. However, a dissociation between modalities was observed in the precentral gyrus, where the motor FRs could be better described by the manipulation model. The emergence of a motor representation based on hand function in the precentral gyrus is likely dependent on the extraordinary motor abilities shown by the 6-fingered hand. It can be hypothesized that the similarity between hand structure and function in 5-fingered subjects may not be simply a byproduct of the mechanical properties of the hand, but the result of an evolutionary process aimed at minimizing the trade-off between effective manipulation abilities, closed-loop somatosensory feedback, and neurocomputational costs. Indeed, the matching between hand structure and function could be exploited and mirrored at the cortical level to align the encoding of sensory and motor information in the precentral and postcentral gyrus. The distorted structure of the hand could emerge from this adapting process. However, while in 5-fingered subjects, the hand structure and function may naturally tend to converge to a joint model, the presence of an additional finger in polydactyly individuals could force them to diverge, preventing the emergence of a common representational model shared between the precentral and postcentral gyrus. In other words, an augmented manipulation ability because of the presence of a supernumerary finger might require additional processing and a more complex organization, in which a dissociation is necessary between the representational geometry of somatosensory and motor information in the precentral gyrus.

In this study, we compared active movements and passive tactile stimulation of the fingers. However, movement of the fingers is inevitably associated with somatosensory feedback, both proprioceptive and tactile. Although we cannot completely rule out that some of our results may be affected by the presence of a tactile component in both the motor and the tactile mapping, we tried to minimize the similarity between the two tasks by applying the tactile stimulation on different locations (palmar and dorsal side of the fingers). Designing a study in which the tactile feedback is abolished in healthy subjects would be impossible; however, future studies may address this limitation by focusing on specific populations, such as deafferented or amputee patients. It should be also considered that tactile stimuli elicited by rapid finger tapping and slow brush stroke follow also partially different pathways of cortical processing (Olausson et al., 2002; Gordon et al., 2013). Since we decided to focus our analyses on the precentral and postcentral gyrus, the possible contribution of tactile processing in other cortical areas to our results could not be assessed.

In conclusion, our study provides a detailed characterization of the representation of the fingers in the sensorimotor cortex. We found distinct motor and tactile FRs in both the precentral and postcentral gyrus, and well-structured anatomic and functional networks involved in the processing and integration of these two modalities. Significant morphologic differences, in terms of somatotopy and cortical volumes, can be identified between the tactile FRs in the precentral compared with the postcentral gyrus, likely reflecting a dissociation in their function, and spatial organizing principles. At the representational level, we found that FRs in different modalities reflect the perceived structure of the hand, which could be the result of an adapting process harmonizing the encoding of hand motor and sensory functions in the precentral and postcentral gyrus. The emergence of such a representational model is also partially independent from the specific hand anatomy in the postcentral gyrus, as this is the case also for the tested polydactyly individual. However, divergent functional-structural properties of the hand, and superior manipulation abilities in this individual, were associated with a motor representation privileging the encoding of the functional properties of the hand in the precentral gyrus.
